# Organic matter sources and flows in tundra wetland food webs

**DOI:** 10.1371/journal.pone.0286368

**Published:** 2023-05-26

**Authors:** Steven P. Plesh, James R. Lovvorn, Micah W. C. Miller

**Affiliations:** 1 School of Biological Sciences, Southern Illinois University, Carbondale, Illinois, United States of America; 2 United States Fish and Wildlife Service, Fairbanks Fish and Wildlife Field Office, Fairbanks, Alaska, United States of America; University of Eldoret, KENYA

## Abstract

Arctic lowland tundra is often dominated by wetlands. As numbers and types of these wetlands change with climate warming, their invertebrate biomass and assemblages may also be affected. Increased influx of nutrients and dissolved organic matter (DOM) from thawing peat may alter the relative availability of organic matter (OM) sources, differentially affecting taxa with disparate dependence on those sources. In five shallow wetland types (<40 to 110 cm deep) and in littoral zones of deeper lakes (>150 cm), we used stable isotopes (δ^13^C, δ^15^N) to compare contributions of four OM sources (periphytic microalgae, cyanobacteria, macrophytes, peat) to the diets of nine macroinvertebrate taxa. Living macrophytes were not distinguishable isotopically from peat that likely contributed most DOM. Within invertebrate taxa, relative OM contributions were similar among all wetland types except deeper lakes. Physidae snails consumed substantial amounts of OM from cyanobacteria. However, for all other taxa examined, microalgae were the dominant or a major OM source (39–82%, mean 59%) in all wetland types except deeper lakes (20‒62%, mean 31%). Macrophytes and macrophyte-derived peat, likely consumed mostly indirectly as DOM-supported bacteria, ranged from 18‒61% (mean 41%) of ultimate OM sources in all wetland types except deeper lakes (38–80%, mean 69%). Invertebrate consumption of microalgal C may often have involved bacterial intermediates, or a mix of algae with bacteria consuming peat-derived OM. High production of periphyton with very low δ^13^C values were favored by continuous daylight illuminating shallow depths, high N and P levels, and high CO_2_ concentrations from bacterial respiration of peat-derived DOM. Although relative OM sources were similar across wetland types except deeper lakes, total invertebrate biomass was much higher in shallow wetlands with emergent vegetation. Impacts of warming on the availability of invertebrate prey to waterbirds will likely depend not on shifts in OM sources, but more on changes in overall number or area of shallow emergent wetlands.

## Introduction

In Arctic lowland tundra of western North America, wetlands can occupy ⁓40% of the landscape (Miller et al. [Unpublished]). Invertebrate food webs in these wetlands are critical to a large and diverse avifauna that migrates there from eastern Asia and from throughout the western hemisphere [1–4]. However, the numbers, extent, and types of these wetlands are changing with climate warming [5, 6]. Moreover, the existing invertebrate diversity and abundance in these wetlands may be threatened by rapid climatic changes in temperature and precipitation, and especially the effects of permafrost thaw on hydrology, water chemistry, and vegetation [7, 8]. Resulting effects on carbon sources, energy flows, and the structure and function of these vital invertebrate communities are poorly known [9–11].

Various invertebrate taxa typically are capable of taking advantage of a range of organic matter sources. However, despite such flexibility, altered relative supply of fresh microalgae, cyanobacteria, macrophyte tissue, and peat-derived dissolved organic matter (DOM) may differentially affect invertebrate taxa with different feeding modes [10, 12, 13]. Macrophyte tissue generally contributes a small fraction of the diet assimilated by most wetland invertebrates [14]. Instead, different taxa variously assimilate fungi, bacteria, microalgae, and their exudates that grow on the surfaces of living and dead macrophyte tissues [15, 16]. Nutrients and DOM leached from macrophytes and algae, as well as DOM entering from the surrounding landscape, can further fuel microbial growth [17, 18].

**Fig 1:**
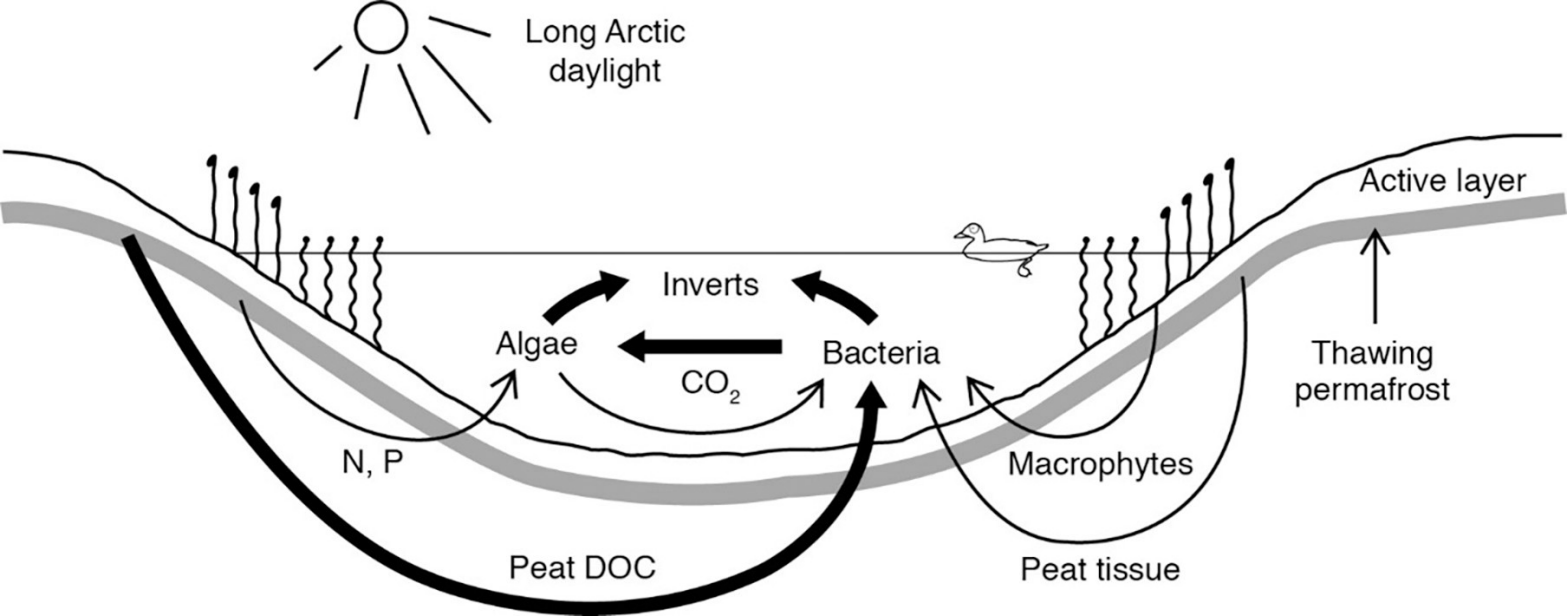
Conceptual model of carbon flow through tundra wetland food webs. The figure includes bacterial respiration of labile peat-derived DOC (dissolved organic carbon) leached from the active layer and adjacent thawing permafrost (region bounded by thick gray line). Resulting CO_2_ facilitates production of microalgae (“algae”, mostly periphyton) with no limitation by nutrients (N, P) or photoperiod.

Climate warming could affect both microalgae and bacteria as foods for invertebrates in tundra wetlands. For example, lowland tundra wetlands of the Alaskan Arctic generally have depths <1 m, very long summer photoperiods, abundant N and P, and high CO_2_ concentrations, which favor high production of periphytic microalgae for both direct herbivory and detrital pathways (Fig 1; [15, 19–22]). Climate-driven increases in the ice-free period will lengthen the growing season for primary producers [9, 23]. At the same time, thawing permafrost landscapes can leach large quantities of organic carbon and nutrients into tundra wetlands [20, 24–26], enhancing bacterial production.

In this study, we used stable isotopes of carbon and nitrogen to investigate the hypothesis that microalgae, cyanobacteria, macrophyte tissue, and peat differed in relative importance as ultimate organic matter sources for different invertebrate taxa in different wetland types of Arctic lowland tundra. We also investigated the relative biomasses of different invertebrate taxa among wetland types, to test the hypothesis that the regional total biomass and community structure of invertebrates available to avian consumers would be affected by changing proportions of different wetland types.

## Methods

### Study area

**Fig 2:**
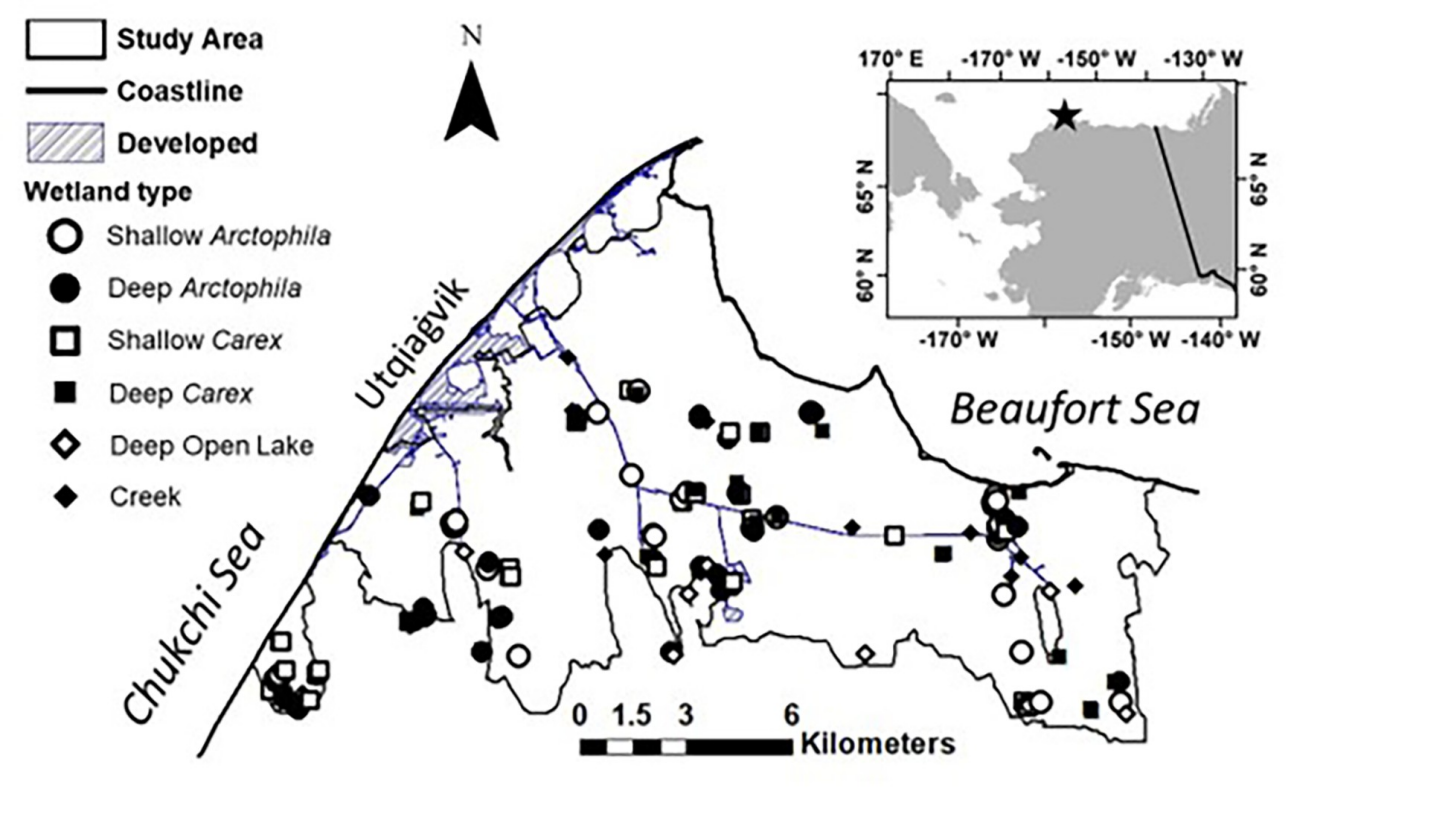
Study area in lowland tundra near Utqiaġvik, Alaska, USA. Map developed using 2002 Quickbird imagery from the National Snow and Ice Data Center [30].

Our study area encompassed 180 km^2^ of lowland tundra near Utqiaġvik, Alaska (71.2906° N, 156.7886° W; Fig 2). Mean temperature is ‒12°C during long winters and 4°C during short summers, with annual precipitation of 11 cm/y. The area has low relief (0‒10 m above sea level), and a thin active (seasonally frozen) layer (30‒40 cm) above continuous permafrost [27]. The landscape is dominated by a mosaic of thaw-lake basins. These basins are formed by creation and merging of flooded ice-wedge polygons and troughs to form shallow lakes, which later drain and revegetate before the cycle (lasting 2000‒5500 y) begins again [28, 29]. These landforms and interstitial tundra host an array of shallow wetlands vegetated mainly by water sedge (*Carex aquatilus*) or pendant grass (*Arctophila fulva*) [6]. *Carex* is emergent, but *Arctophila* can be emergent or submersed depending on water depth. *Carex* meadows also dominate uplands around the wetlands [27, 29]. In this area, tundra wetlands occupy a proportion of the landscape that is probably comparable to that in lowland tundra throughout the extensive Alaskan Arctic Coastal Plain (Miller et al. [Unpublished]).

In the spring, frozen ground prevents penetration of snowmelt, which flows mostly into larger stream channels which flood seasonally. In summer and fall, the low topographic relief limits lateral run-off of rain as well as lateral transport of water through the active layer of soil, resulting in ponding at the surface. Thus, water budgets of the wetlands are driven mainly by direct summer precipitation and evapotranspiration [31]. Wetland sediments are generally flocculent and highly organic (~82% organic C). Microbial primary producers (mostly green algae and cyanobacteria in roughly similar proportions) are mostly attached to sediment and detritus particles, with relatively little production in the shallow water column [32]. Benthic algae generally do not form algal mats owing to bioturbation, and the main control on benthic algal biomass appears to be light limitation by mixing of algae into the sediments [32].

### Sample collection and processing

We sampled six wetland types (based mostly on the classification of Bergman et al. [1]) characterized by depth and dominant macrophyte species: Shallow *Arctophila* (*n* = 14 wetlands), Deep *Arctophila* (*n* = 10), Shallow *Carex* (*n* = 11), Deep *Carex* (*n* = 7), Streams (*n* = 6), and Deep Open Lakes (*n* = 4). Wetlands dominated by *Arctophila* or *Carex* were termed “deep” if depth exceeded 40 cm, although depth was typically <110 cm. Deep Open Lakes were defined as having maximum depth >150 cm and surface area >1 ha, typically with no macrophytes present. Deep Open Lakes were generally >2 m deep, with some probably up to 8 m deep; they typically did not freeze in their entirety to the bottom in winter and some contained fish (mainly ninespine stickleback, *Pungitius pungitius* [33]). However, all of our sampling of Deep Open Lakes was in littoral areas <1 m deep that were accessible by wading. Streams were shallow, slow-moving, beaded streams between basins or between basins and coastal sloughs, usually with substantial overbank flooding during the spring. Equivalence of these different wetland types with the classification of Cowardin et al. [34] was documented by Derksen et al. [35].

We collected macrophyte and invertebrate samples in June and July of 2013, 2017, and 2018. In all three years, we used a pole-mounted, triangular sweep-net with 500-μm mesh (Wildco, Yulee, Florida, USA). In 2013, samples were collected for stable isotope analyses, but were not suitable for estimating invertebrate densities. One net sample was collected by scraping through the upper sediments, and the second sample by sweeping the net through vegetation (*Carex* or *Arctophila*); the two samples were combined into a single aggregate sample per wetland. In 2017 and 2018, we obtained quantitative net samples within stands of macrophytes at two different locations per wetland. At each location, we swept the net horizontally for 2.5 m through the water column 10 cm below the water surface to capture invertebrates found within the vegetation. Also in 2017 and 2018, at four different locations per wetland, we pressed an acrylic corer (inside diameter 5.2 cm, length 50 cm) through the sediments until it encountered the subsurface ice layer. If water depth exceeded 50 cm, a valve attached to the corer was closed to create a vacuum which assisted extraction of the core. The top 10 cm of the resulting core was filtered through a 500-μm sieve to retain invertebrates. If the corer encountered a frozen layer <10 cm deep in the sediment, biomasses per unit area were corrected based on volume sampled. All plant and invertebrate samples were frozen for later processing.

In the laboratory, invertebrate samples were thawed, sorted, and counted. Insects identified from our samples included larval forms of Diptera (Chironomidae and Tipulidae), Plecoptera, Trichoptera, and Coleoptera (mainly Dytiscidae). Other invertebrate taxa were Acari, Crustacea (mostly Copepoda with some Notostraca and Daphnidae), Oligochaeta, and Gastropoda of the family Physidae. Identification to lower taxonomic levels for some taxa was hindered by sample degradation during freezing. Macrophyte tissue was identified as *Carex* or *Arctophila*.

We did not separate larvae of Chironomidae into predatory (subfamily Tanypodinae) vs. non-predatory taxa. Butler et al. [36] reported that Tanypodinae averaged 3.8% (range 1.1 to 6.4%) of numbers of all chironomid larvae in three wetlands in our study area. In 12 wetlands in our study area, Lougheed et al. [11] found Tanypodinae to comprise 6.5% of total chironomid numbers in 1971‒1973 and 2.2% in 2007‒2009. Moreover, various Tanypodinae are facultative predators, often consuming partly or mainly detritus [37–39]. Given the small fraction of Tanypodinae and their often mixed feeding modes, we analyzed all Chironomidae as a single taxon.

### Invertebrate masses and stable isotope analyses

For analyses of μg C per individual (S3 Table), samples for each taxon were pooled across wetland types to obtain large enough sample masses. For biomasses of each taxon within each wetland type and sample type (cores vs. sweeps), means were calculated across wetlands of each type.

For isotopic analyses, obtaining large enough masses required pooling of samples within taxon and wetland type. Specimens from each invertebrate taxon were counted, aggregated within wetland, oven-dried for 24 h at 55°C, ground with a mortar and pestle, and fumed with 10% HCl for 20 min to remove inorganic carbonates that might confound δ^13^C values of OM. These samples were then placed into folded, precombusted glass-fiber filters (Whatman GF/F, Cytivia, Marlborough, MA, USA). All individuals of a given taxon in samples from a given wetland were aggregated in a single filter. Lipids have lower δ^13^C values than proteins or carbohydrates, so that the whole-body δ^13^C values of the same organism can vary appreciably depending on its current state of lipid storage. To eliminate this variation, Post et al. [40] recommended lipid extraction when lipid content varies among consumers or between consumers and prey endmembers, and when δ^13^C values between endmembers differs by <10‒12‰. In our study, lipid content ranged from 3 to 23% among consumer taxa, and differences in δ^13^C values among endmembers were in most cases <10‰ (Table 1). Accordingly, we extracted lipids from all invertebrate samples. The filters with samples were immersed in 99% petroleum ether for 72 h to remove lipids, with the ether replaced every 8 h [41].

**Table 1:** Means and SE of stable isotope values (δ^13^C, δ^15^N) of endmembers for organic matter sources used in mixing models, based on measured and reported values (S1 Table).

Endmember	δ^13^C (‰)	δ^15^N (‰)	References
Algae	‒38.0 ± 2.5*	2.4 ± 0.2	[42]
Macrophytes	–29.5 ± 0.1	1.8 ± 0.2	Collected *in situ*
Peat	–28.2 ± 0.2	‒0.8 ± 0.4	[43–45]
Cyanobacteria	–22.6 ± 1.5	1.3 ± 0.5	[43, 46, 47]

*Reported mean of ‒37‰ was decreased by 1‰ to encompass most values measured in primary consumers (see text).

After lipid extraction, invertebrate samples were oven-dried again for 48 h at 55°C and then ground with a mortar and pestle. Vegetation samples were dried and homogenized with a household coffee grinder. Samples from each filter (with varying total masses) were stored in sealed glass vials until being subsampled for analyses of δ^13^C and δ^15^N at the University of Wyoming Stable Isotope Facility, via a Costech 4010 elemental analyzer (Costech Scientific, Valencia, CA, USA) coupled with a Thermo Delta Plus XP isotope-ratio mass spectrometer (Thermo Fisher Scientific, Waltham, MA, USA). Stable isotope values are expressed in units of parts per thousand (‰), as the ratio of heavy to light isotope relative to a standard. This quantity is calculated by the equation δ*X* = [(*R*_sample_/*R*_standard_)– 1] × 1000, where *X* is ^13^C or ^15^N and *R* is the ratio of ^13^C/^12^C or ^15^N/^14^N. Values are reported with respect to Vienna PeeDee Belemnite for δ^13^C and atmospheric N_2_ for δ^15^N. Measurements of internal laboratory standards (peptone) yielded a precision of 0.1% for both δ^13^C and δ^15^N values.

### Endmembers

For endmembers (organic matter sources) in mixing models, we used stable isotope values from our own samples for macrophyte tissue, and means among values reported in the literature for periphytic (benthic and epiphytic) microalgae, peat, and cyanobacteria (Table 1 and S1 Table). Obtaining a pure and consistent isotopic signal for periphytic algae from field samples can be notoriously difficult. Especially in flocculent, highly organic sediments, the periphytic layer is typically a complex community that includes fungi, bacteria, protists, organic tissue fragments, exopolymer secreted by both algae and bacteria, and DOM sorbed to and diffusing into the periphytic matrix from the water column [48, 49]. Indeed, algal cells are typically a minor component of periphytic carbon, averaging only 8% over a range of substrate types [50]. Thus, measurements of the bulk isotopic composition of periphytic samples often cannot be used reliably to represent that of primary producers alone, being too enriched in ^13^C. In our study area, broadscale sampling of periphytic communities in wetlands with flocculent, highly organic sediments, and active bioturbation that generally prevents formation of algal mats [32], was unlikely to yield accurate isotopic values for periphytic algae alone. Such measurements would not encompass the lower δ^13^C signatures for algivores and thus would not yield a suitable endmember. Owing to these technical challenges, similar studies have often used literature values of algae scraped from hard surfaces as the best proxy for periphyton in soft sediments [e.g. 51]. The available value for such periphyton samples in our region was ‒37‰ δ^13^C for streams in areas of ice-rich permafrost in northwest Alaska [42] (Table 1 and S1 Table).

**Fig 3:**
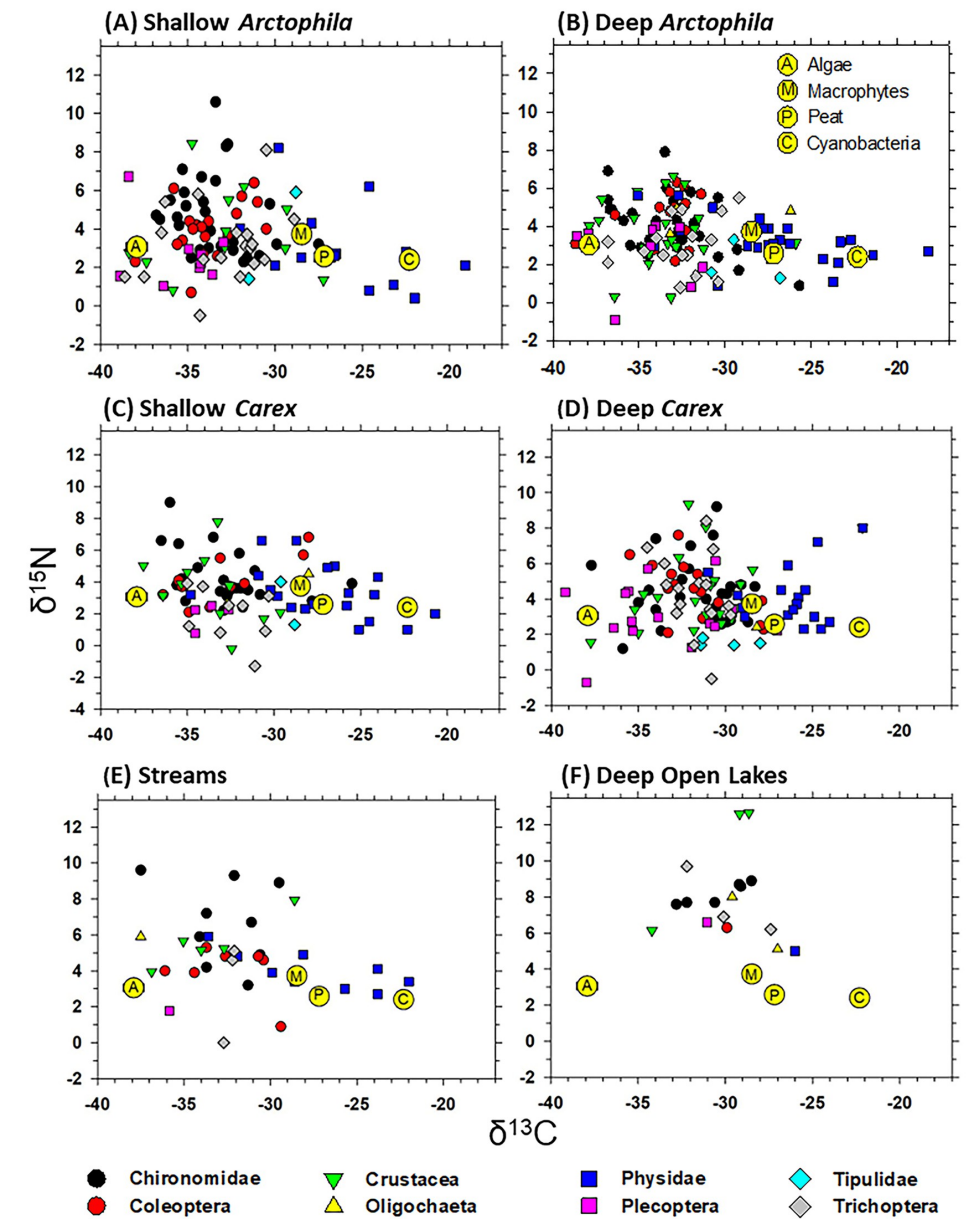
Stable isotope scatterplots for each wetland type. Endmembers for organic matter sources with trophic discrimination factors applied (Table 2) are also plotted (yellow circles). (**A**) Shallow *Arctophila*, (**B**) Deep *Arctophila*, (**C**) Shallow *Carex*, (**D**) Deep *Carex*, (**E**) Streams, and (**F**) Deep Open Lakes.

Moreover, the guts of deposit-feeders typically contain both recognizable cells (e.g. diatoms, cyanobacteria) and unidentified particles termed “amorphous detritus” which are thought to include flocculated exopolymer secreted by algae and bacteria. Such mixtures in gut contents indicate ingestion of multiple components of the periphytic community [15, 49]. Literature values indicated that algae had by far the lowest δ^13^C value of the potential organic matter sources for wetlands in our study (Table 1 and S1 Table). Therefore, as has been done in similar cases with variable fractionation by microbes and uncertain selectivity by deposit-feeders, we considered the most reliable pure endmember for benthic microalgae to be the most extreme (most depleted in ^13^C) value among the primary consumers [cf. 52, 53]. In mixing models we used a δ^13^C value of ‒38‰, which encompassed almost all of the most depleted values for invertebrates (Fig 3) and was consistent with the literature value (‒37‰).

**Table 2:** Trophic discrimination factors (TDFs, Δ^13^C, Δ^15^N) for macroinvertebrate consumers relative to organic matter sources (endmembers), and trophic route involved in the fractionation, in tundra wetlands. Literature survey for the TDFs is in S2 Table. Values in parentheses are for endmembers (Table 1) plus the corresponding TDFs; resulting values are plotted in Fig 3.

Organic matter source and trophic route	Δδ^13^C (‰)	Δδ^15^N (‰)
Algae: fresh algal cells directly to invertebrates	0.09 (‒37.91)	0.66 (3.06)
Periphytic community: fresh algae or phytodetritus to bacteria and protists to invertebrates	1.12 (‒36.88)	1.70 (4.10)
Macrophytes: macrophyte tissue to fungi to invertebrates	1.03 (‒28.47)	1.93 (3.73)
Peat: peat tissue to DOM to bacteria and protists to invertebrates	1.02 (‒27.18)	3.38 (2.58)
Cyanobacteria: cyanobacteria directly to invertebrates	0.3 (‒22.3)	1.1 (2.4)

For peat in tundra environments, the isotopic signature can vary with depth through the active layer and the upper boundary of permafrost [42, 54]. However, within the active layer, permeability to lateral porewater movement decreases sharply with increasing depth [55, 56], and in our study area there is mostly laminar horizontal flow with little vertical mixing among depth layers [31]. Given that the active layer above impermeable permafrost deepens slowly as it thaws over several months [31], pore water moving laterally will collect DOM mostly from a relatively shallow layer of peat. In fact, most DOC entering shallow tundra wetlands is derived from this near-surface layer of more recently deposited organic matter [57].

This upper layer of peat, termed the acrotelm, differs from deeper layers in that it has much higher hydraulic conductivity, is periodically aerated, is rich in peat-forming bacteria and other microorganisms, and includes both living and dead plant material [58]. In peat formed mainly from *Carex* as around wetlands in our study area, the peat consists mostly of living and dead *Carex* roots that penetrate downward through the acrotelm [59, 60]. Isotopic measurements at five sites across an Icelandic landscape of peat derived mainly from *Carex* [45], as well as from six sites across our study region, yielded standard errors of only 0.21‰ in δ^13^C and 0.38‰ in δ^15^N (Table 1 and S1 Table). Given these very low variances among different areas for the same habitat, we considered the means and SE from literature values for peat to be suitable for our mixing models.

Cyanobacteria presented similar challenges as periphytic microalgae. Consequently, we used published values from a nearby site where samples could be collected from cyanobacterial mats, and from lakes in the Alaskan Arctic and a temperate region where deeper water allowed filtering adequate samples from the water column.

Note that for all endmembers derived from literature surveys, associated variances (S1 Table) are accounted for in mixing model software (MixSIAR) used to compare individual samples to different OM matter sources. When considering the range of possible values from the literature, there was clear separation of the endmember values (Fig 3) and good convergence of Monte Carlo simulations in mixing models (see section on Mixing models).

Because methane-oxidizing bacteria are strongly depleted in ^13^C, they can have important effects on the δ^13^C values of consumers [61]. However, because local measurements suggest that the role of methanotrophs in these systems is minimal (see details in the Discussion), we did not include an endmember for methane-oxidizing bacteria.

### Trophic discrimination factors

Mixing models require trophic discrimination factors (TDFs), or the change (fractionation) in isotopic values that occurs between ingestion and incorporation into consumer tissues (written as Δ^13^C and Δ^15^N). General averages of TDFs are commonly used (e.g. ≤1 ‰ for Δ^13^C and 3.4 ‰ for Δ^15^N, [52]). However, these values often do not apply to consumption by heterotrophic microbes or to metazoan consumption of those microbes or algae. In our study, TDFs between basal organic matter sources and deposit-feeding macroinvertebrates were based on a survey of published values (Table 2 and S2 Table).

When peat layers in surrounding tundra thaw, the organic matter in dead plant tissue is metabolized and fractionated by bacteria to be released as DOM [62]. This DOM is then carried by pore water diffusing through subsurface material into the wetlands. Once in the wetlands, peat-derived DOM can be further fractionated by photolysis [63], and can sorb to the periphytic layer and diffuse into that matrix for use by bacteria [48, 64]. The activity of bacteria bound to surfaces is generally far greater than that of unbound bacteria in the water column [48]. By the time these bacteria or their exudates are subsequently ingested by deposit-feeding macroinvertebrates, organic molecules of the original peat have been fractionated multiple times. We acknowledged this repeated fractionation in estimating the TDFs between peat tissue and deposit-feeding macroinvertebrates (Table 2 and S2 Table).

The bacterial fraction of the periphytic community can ingest only DOM, which may be derived from lysed cells or exudates of algae, from leachates of macrophytes [65], or from peat DOM leaching into the wetland from surrounding tundra. Although predation on bacteria by protists may insert another trophic level, fractionation by protists consuming bacteria appears to be minimal [66, 67].

Peat OM enters the wetlands almost entirely as DOM, and bacteria generally can consume OM only as DOM [48]. Thus, we assumed that almost all peat DOM is ingested by detritivores in the form of bacteria or their flocculated exudates. Fresh algal cells can be consumed directly by detritivores, whereas we assumed that algal exudates and dead algal cells are assimilated by detritivores mainly in the form of bacteria [48]. These assumptions are consistent with the prevailing view that primary production in freshwater ecosystems, especially those dominated by detritivores, enters metazoan food webs mainly as detritus via bacterial and fungal intermediates [68, 69]. Fractionation of N isotopes between fresh algal cells and invertebrate consumers tends to be quite low, whereas we expect fractionation between peat DOM and dead algae to detritivores to be substantially higher (including bacterial or fungal intermediates) (Table 2).

We assumed that macrophyte tissue (litter) is assimilated by detritivores mainly as bacteria and especially as fungi growing on the tissue (Table 2) (cf. [16]). Particulate matter composed of plant tissues may be largely refractory to invertebrate digestion [70, 71], but bacteria colonizing the surface of particulate matter can provide readily accessible nutrients for invertebrate consumers [72]. Fungal biomass can far exceed bacterial biomass in attached communities, representing 70‒99% of microbial biomass on decomposing leaves (including emergent macrophytes) in fresh waters [73, 74], and 70% of total microbial carbon in sediments of a wetland in our study area [75]. Unfortunately, the role of fungi in detritivore diets is poorly studied in aquatic systems.

### Mixing models

Although TDFs for δ^15^N were assigned for initial display purposes (Fig 3), very wide variation in δ^15^N indicated that variable numbers of trophic levels within the microbial loop confounded selection of consistent TDFs for δ^15^N [cf. 53]. Consequently, δ^15^N values were of limited value for discriminating organic matter sources, so we used single-isotope models for δ^13^C only. We used the Bayesian mixing model software MixSIAR [76] to estimate diets of each invertebrate taxon within each wetland type. Proportions of organic matter sources for different invertebrates were unknown, so we chose an uninformative prior for analyses. We ran models with the following Markov Chain Monte Carlo parameters: 3 chains; 300,000 samples; a 200,000 sample burn-in; and retention of each 100^th^ sample to minimize autocorrelation (a “long” run in MixSIAR).

### Biomass estimates

We determined the carbon content of invertebrates from subsamples prepared for stable isotope analyses. We divided the C biomass by the number of individuals per subsample to yield an estimate of C biomass per individual for each taxon (S3 Table). The latter estimate was then multiplied by counts in field samples to yield g C m^‒2^ for each taxon for cores and net sweeps separately in each wetland. We then calculated mean biomass in cores and sweeps for each invertebrate taxon among wetlands within each wetland type. Tipulidae and Oligochaeta were not included in biomass analyses. Tipulidae were generally rare in all wetland types and were not detected in Deep Open Lakes or Streams. Although samples of Oligochaeta were adequate for stable isotope analyses, degradation of Oligochaeta by freezing prevented accurate biomass determinations.

We used PRIMER version 7 with the PERMANOVA+ add-on to test for differences in biomass among wetland types for core and sweep samples separately [77]. We transformed the data by log (*x* + 2) before constructing Bray-Curtis dissimilarity matrices. For comparing invertebrate communities often dominated by a few taxa, this transformation is recommended to down-weight dominant taxa and enhance consideration of taxa with lower total biomass [78]. Using wetland type and individual wetland as factors, we ran a Permutational Analysis of Variance (PERMANOVA) with pairwise comparisons of wetland type for each invertebrate taxon. For comparisons with less than 100 unique permutations, we conducted Monte Carlo tests to obtain pseudo-*F* statistics and permutation-*p* values.

## Results

Almost all taxa in this mainly deposit-feeding community appeared to reflect a mix of mostly periphytic algae, macrophytes, and peat as ultimate organic matter sources, with Physidae snails also ingesting appreciable cyanobacteria (Fig 3; for means and SE see S4 Table). We initially considered macrophytes and peat as separate endmembers; however, these OM sources were too similar in δ^13^C values to be discriminated reliably in mixing models. The upper layer (acrotelm) of *Carex*-derived peat that likely yields most DOM leached into tundra wetlands is composed mostly of both living and dead *Carex* roots, perhaps with some above-ground macrophyte litter that is rapidly decomposed [55, 56, 59, 60]. Consequently, we combined macrophytes and peat in mixing models for all wetland types.

### Organic matter sources of invertebrate taxa by wetland type

A single-isotope model cannot discriminate more than two endmembers. In such cases, one must use only the two most likely endmembers suggested by the isotope biplots [79], which were periphytic microalgae and macrophytes-peat (Fig 3). Cyanobacteria were important only to Physidae snails, and to a lesser extent Oligochaeta and Tipulidae, so these taxa were excluded from single-isotope models.

The continuous distribution of δ^13^C values within deposit-feeders (Fig 3) suggested variation in fractionation of algal inputs (multiple trophic levels) within the microbial loop, variation in dietary proportions of algae vs. macrophytes-peat, or both. Our mixing models could not distinguish these possibilities, attributing all intermediate values to mixtures of the endmembers rather than to variable fractionation of the algal endmember.

**Fig 4:**
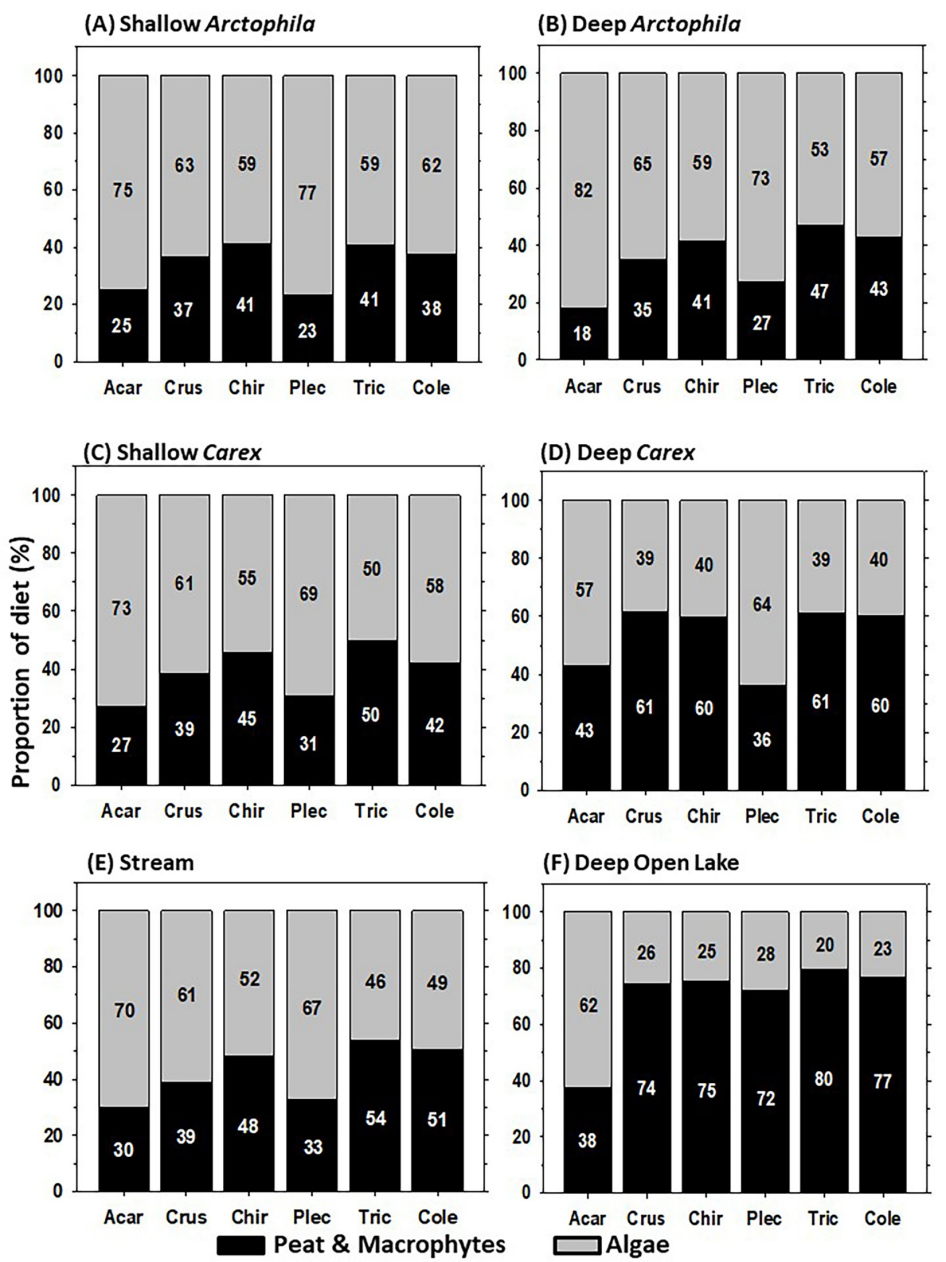
Relative contributions (mean percentages) of periphytic microalgae and of peat and macrophytes combined to diets of invertebrates across six wetland types. For data values see S6 Table. Acar = Acari, Crus = Crustacea, Chir = Chironomidae, Plec = Plecoptera, Tric = Trichoptera, Tipu = Tipulidae, Cole = Coleoptera, Olig = Oligochaeta, Phys = Physidae). (**A**) Shallow *Arctophila* (*n =* 34), (**B**) Deep *Arctophila* (*n* = 26), (**C**) Shallow *Carex* (*n* = 36), (**D**) Deep *Carex* (*n* = 40), (**E**) Streams (*n* = 11), and (**F**) Deep Open Lakes. Asterisks (*) indicate taxa that were not detected in a given wetland type.

Results of mixing models (Fig 4) indicated that in Shallow and Deep *Arctophila* and Shallow *Carex* wetlands, microalgae were the dominant ultimate source of organic matter for all invertebrate taxa examined (50‒82%, mean 64%). (Statistics on mixing model convergence are in S5 Table, and means and SE of model estimates of the percentages of organic matter sources are in S6 Table.) In the same wetlands, macrophytes-peat accounted for only 18‒50% (mean 36%) of OM inputs for these invertebrates. In Deep *Carex* wetlands, microalgae represented 39‒64% (mean 46%) of OM inputs in invertebrates, with slightly larger contributions from macrophytes-peat (36‒61%, mean 54%) (Fig 4D). In Streams, microalgae accounted for 46‒70% (mean 58%) of OM inputs, compared to contributions of 30‒54% (mean 43%) from macrophytes-peat (Fig 4E). In contrast to the shallower wetland types, invertebrates in Deep Open Lakes reflected mainly macrophytes-peat as the dominant carbon source (38‒80%, mean 69%), with lesser contributions of microalgae (20‒62%, mean 31%) (Fig 4F). Inspection of Fig 3 indicates that cyanobacteria, which were not included in our two-source mixing models, were probably a minor dietary component for all taxa except Physidae snails.

### Invertebrate biomass

**Fig 5:**
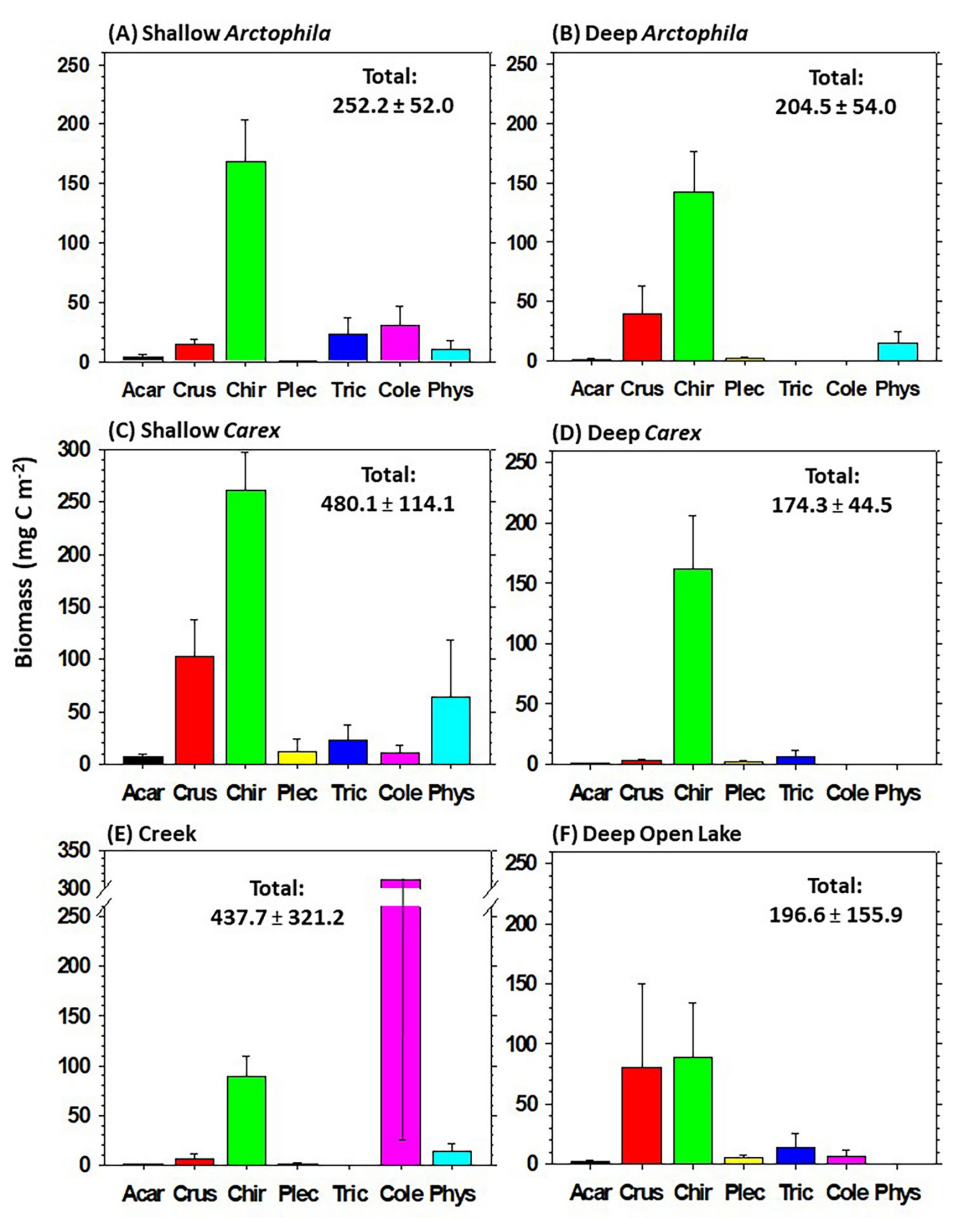
Mean ± SE of invertebrate biomass (mg C m^‒2^) in benthic cores across six wetland types. (Acar = Acari, Crus = Crustacea, Chir = Chironomidae, Plec = Plecoptera, Tric = Trichoptera, Cole = Coleoptera, Phys = Physidae,). (**A**) Shallow *Arctophila* (*n* = 14), (**B**) Deep *Arctophila* (*n* = 10), (**C**) Shallow *Carex* (*n* = 11), (**D**) Deep *Carex* (*n* = 7), (**E**) Streams (*n* = 6), and (**F**) Deep Open Lakes (*n* = 4). Total biomass of all invertebrates is annotated in each panel. Numerical values and results of statistical tests are in S7 Table.

Although patterns of organic matter sources were mostly similar among invertebrate taxa in different wetland types, total and relative biomasses among taxa in cores were highly variable among wetland types (Fig 5; for numerical values and results of statistical tests, see S7 Table). Chironomidae had consistently high or the highest biomass of all taxa. Coleoptera (mainly Dytiscidae) had exceptionally high biomass in at least some Streams (although with very high variance among wetlands of this type), but relatively low or negligible biomass in other wetland types. Crustacea had very low biomass in some wetland types, and moderate biomass in others. Acari always had negligible biomass, while Plecoptera, Trichoptera, and Physidae generally exhibited low and variable biomasses among wetland types.

**Fig 6:**
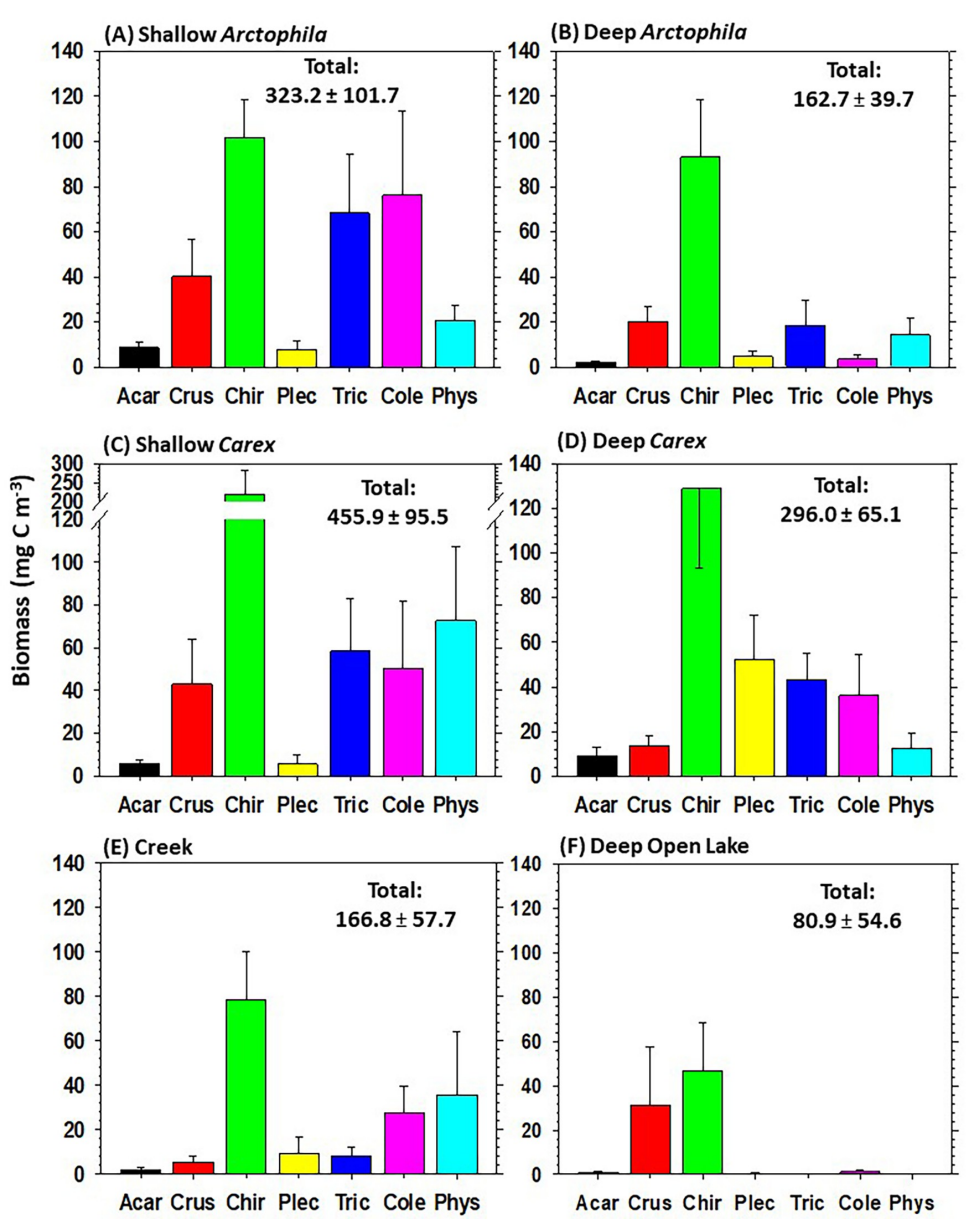
Mean ± SE of invertebrate biomass (mg C m^‒3^) in net sweeps through emergent vegetation across six wetland types. Acar = Acari, Crus = Crustacea, Chir = Chironomidae, Plec = Plecoptera, Tric = Trichoptera, Cole = Coleoptera, Phys = Physidae). (**A**) Shallow *Arctophila* (*n* = 12), (**B**) Deep *Arctophila* (*n* = 10), (**C**) Shallow *Carex* (*n* = 11), (**D**) Deep *Carex* (*n* = 7), (**E**) Streams (*n* = 6), and (**F**) Deep Open Lakes (*n* = 4). Total biomass of all invertebrates is annotated in each panel. Numerical values and results of statistical tests are in S8 Table.

Net sweeps in the water column within emergent stands yielded more consistent and even representation of invertebrate taxa (Fig 6; for numerical values and results of statistical tests, see S8 Table). Chironomidae were again the dominant taxon in all wetland types, having especially high biomass in Shallow *Carex*. Trichoptera and Coleoptera were relatively more important to total biomass in sweeps than in cores for most wetland types. Shallow *Arctophila* and Shallow *Carex* had 2‒3 times the total invertebrate biomass of Deep *Arctophila*, and Shallow *Carex* had 54% higher biomass than Deep *Carex*. The *Carex* wetlands had 41 to 89% higher total biomass than *Arctophila* wetlands of comparable depth. Although Streams had relatively high biomass of Chironomidae, total biomass of invertebrates was relatively low. Except for Crustacea (mostly Copepoda with some Notostraca and Daphnidae), the biomass of invertebrates was generally far lower in the littoral zones of Deep Open Lakes than in the other wetland types.

## Discussion

Our analyses indicate that the generally low δ^13^C values of the deposit-feeding community reflect consumption of a mix of microalgae, of bacteria consuming microalgae or algal exudates, and of bacteria consuming DOC derived from macrophytes-peat. Expected trends in these organic matter sources with climate change suggest that their availability will remain high or increase, with little impact on the structure of invertebrate communities. However, the different wetland types varied widely in total biomass of invertebrates. Thus, ongoing and expected changes in relative occurrence of different wetland types may have important effects on invertebrate prey availability to avian consumers.

### Methane oxidation vs. fractionation during DIC uptake

For invertebrates with δ^13^C values intermediate between microalgae and macrophytes-peat (Fig 3), it is also possible that their carbon sources included a mixture of macrophytes-peat with relatively high δ^13^C values, and of methane-oxidizing bacteria with very low δ^13^C values. Indeed, our values of δ^13^C in deposit-feeding invertebrates were often in the “zone of contention” (‒40 to ‒30‰), meaning that these values might or might not represent dietary mixtures of moderately depleted foods with methanotrophic bacteria strongly depleted in ^13^C [80]. However, several lines of evidence suggest that the latter possibility is less likely in our tundra wetlands.

In wetlands in our study area, Throckmorton et al. [54] reported that most subsurface methane was routed upward through the stems and leaves of emergent plants, mainly bypassing the potential for methane oxidation. As a result, only 2.5% (July) and 0.8% (September) of methane produced was consumed by methanotrophic bacteria, accounting for <0.1% of dissolved inorganic carbon (DIC) production. Instead, DIC was produced mostly via respiration by non-methanotrophic bacteria. The δ^13^C value of DIC in porewater at the surface of the active layer was ‒12‰ [54], further indicating that little methane oxidation was occurring near the sediment surface. Methane oxidation has been important mainly in deeper, stratified lakes with anoxic sediments and bottom water [61]; however, anoxia favoring some methane oxidation can occur in organic, subsurface sediments of shallow lakes (< 2 m deep), perhaps especially those with dense macrophytes [81, 82]. In contrast, in our area and others with polygonal tundra, macrophytes appear to channel most methane past the zone of methane oxidation [23, 54, 83]. Moreover, a survey of 87 temperate lakes indicated that appreciable consumption of methanotrophic bacteria by chironomid larvae occurred only when oxygen concentrations near the sediment surface fell below about 2 mg O_2_/L [61, 84]. Our tundra wetlands were generally <1 m deep with perpetually windy conditions, and past measurements in three wetlands yielded a minimum of 3.5 mg O_2_/L within an emergent stand of *Carex aquatilis* [85]. Together with the findings of Throckmorton et al. [54], these aspects suggest that our vegetated tundra wetlands supported little biomass or production of methanotrophic bacteria, and yielded little respired CO_2_ from methanotrophs available for uptake by benthic algae [61].

Alternatively, the low δ^13^C values in deposit-feeders could result merely from selective algal uptake of DIC with low δ^13^C value, given the very high partial pressure of CO_2_ (pCO_2_) in these wetlands. For mean summer temperature during mid-day at Barrow (⁓7.2°C), the CO_2_ solubility coefficient (*K*_o_) is 0.059 mol/kg atm. For a pCO_2_ of 1.3336 atm measured in local wetlands (Table 2 in [22]), CO_2(aq)_ = pCO_2_ / *K*_o_ = 22.6 μmol/L. Based on eqn (3) of Lennon et al. [86], concentration-dependent fractionation between CO_2(aq)_ and HCO_3_^‒^ during photosynthesis by microalgae is estimated as ‒ [25.4 × CO_2(aq)_] / [3.7 + CO_2(aq)_] = ‒21.8‰. Relative to δ^13^C values of ambient DIC at the sediment surface of ‒12‰ [54], benthic microalgae should have δ^13^C values of about ‒33.8‰, and primary consumers about ‒32.8‰ (assuming Δ^13^C ≈ 1‰). Given errors and assumptions in these estimates, isotopic fractionation during microalgal uptake of DIC at such high CO_2_ concentrations appears adequate to explain most if not all of the values observed in deposit-feeders, without invoking consumption of methanotrophic bacteria. The main taxa reported to consume methanotrophic bacteria are chironomid larvae, in particular those living in irrigated tubes containing oxygenated water adjacent to anoxic surrounding sediments [61]. However, in our wetlands, diverse deposit-feeding invertebrates (Crustacea, Plecoptera, Trichoptera) overlapped in δ^13^C values with chironomids (Fig 3), suggesting that the most often-cited mechanism supporting consumption of methanotrophs was not necessary to produce the observed values.

In summary, we believe that direct algivory, and consumption of bacteria that have ingested algae, their exudates, or DOC leached from macrophytes-peat, are the most important carbon sources at the base of invertebrate food webs in these tundra wetlands. Consumption of methane-oxidizing bacteria can be appreciable in other areas, but is highly variable within and among systems [61] and in our case appears to be much less important. We note that Physidae snails appeared to be unique in consuming substantial amounts of OM from cyanobacteria (Fig 3).

### Effects on endmember values

Across invertebrate taxa, δ^13^C values were quite low compared to literature values for available organic matter sources except microalgae (cf. Fig 3, Table 1 and S1 Table). Lipids exhibit more negative δ^13^C values compared to other tissues [40], but extraction of lipids from our samples prevented this effect. A microalgal endmember of –38‰ was needed to encompass most values in our samples (Fig 3). Values for biofilm as low as ‒40‰ in streams flowing through permafrost areas south of our study sites [42] (Table 1 and S1 Table) indicate that such a low endmember value is reasonable.

In these tundra wetlands mostly <1 m deep with almost continuous daylight and very high levels of N and P (Fig 1) [19–21, 87, 88], microalgae (mostly periphyton) likely have very high productivity and were an important carbon source for most taxa (Figs 3 and 4). The extraordinarily high pCO_2_ in these wetlands (up to 5000 μatm, [22]) may result in proportionately strong selection of the light isotope of C during uptake of DIC by microalgae (see previous section), leading to very low δ^13^C values in microalgal consumers. In local wetlands, CO_2_ levels in direct proximity to benthic microalgae were driven mainly by bacterial respiration [54], while very high DIC levels may in fact reduce diffusion of atmospheric CO_2_ through the water column to the sediment surface [57]. In local wetlands in late July, DOC concentrations increased by almost 60% (about 11.6 to 18.5 mg L^‒1^) and pCO_2_ by 228% (about 688 to 1568 μatm) from 1971 to 2009‒2013, apparently associated with increased thawing of surrounding tundra [22] (see also [62, 89]). As warming and tundra thawing has continued since that time, this scenario may explain the low δ^13^C values in our invertebrates.

Within periphyton communities, cyanobacteria generally have substantially higher δ^13^C values than do other primary producers [90]. Cyanobacteria concentrate inorganic carbon more efficiently than other groups, and thus can take up higher amounts of HCO_3_^‒^ relative to CO_2_ when CO_2_ is less available [91]. Because the δ^13^C value of HCO_3_^‒^ is higher than that of dissolved CO_2_, this difference may explain the generally higher δ^13^C value in cyanobacteria (Fig 3) [90]. However, except when CO_2_ is in short supply as during intense blooms or in still waters, cyanobacteria normally take up CO_2_ rather than HCO_3_^‒^ [92]. Another possible explanation for the much higher δ^13^C value in cyanobacteria than in eukaryotic microalgae is that some cyanobacteria are known to consume organic compounds as an alternative nutrient source when such compounds are abundant [93, 94]. Feng et al. [95] noted that N fixation is an energy demanding process, and that availability of labile, energy-rich C substrates to cyanobacteria eliminates the cost of C fixation to fuel the cost of N fixation, thereby allowing maximum growth. The value we used for δ^13^C of cyanobacteria was a measurement for mats of *Nostoc* spp. in our study region [43], and additional measurements are needed as pCO_2_ increases in these wetlands [22].

The porosity and hydraulic conductivity of peat generally declines sharply at depths below 10‒20 cm, lateral flow through the peat in our area is mostly laminar, and deepening of the active layer above impermeable permafrost with thawing over the summer is gradual [31, 55]. Accordingly, much of the DOM leached into wetlands likely comes from relatively recently deposited plant material in the peat acrotelm, despite increasing contributions from thawing of the upper layer of permafrost containing older carbon [57]. Given that the acrotelm in peat formed mainly from *Carex* roots typically contains both living and dead tissue, we could not distinguish macrophytes from peat in surrounding tundra as OM sources for macroinvertebrates. ^14^C dating of peat from the active layer and upper layer of permafrost might help discriminate the supply and uptake of increased DOM from thawing permafrost vs. increased DOM from the active layer due to a longer annual period of thawing [42, 57, 64, 96].

An important caveat to our study is that the endmembers of epiphytic microalgae, peat, and cyanobacteria were based on averages of literature values, including those from in and near our study area, as opposed to direct measurements for our study sites (S1 Table). Detailed justification for this approach is provided in the *Endmembers* section of the Methods. Although we doubt that directly measured values would have differed substantially or shown greater repeatability, future studies should attempt to overcome the challenges of sampling in ways that reflect more accurately the local isotopic values of foods available to and selected by various invertebrate consumers.

### Patterns of variation in invertebrates

The δ^13^C values of most invertebrate samples fell in a range between the very low endmember for fresh microalgae and the much higher endmembers for macrophytes and peat (Fig 3). These intermediate values could represent consumption of a combination of these materials, as mixing models between two endmembers are expected to indicate. However, these values might also reflect ingestion of microalgal phytodetritus that had been reworked and fractionated to varying degrees by bacteria and macrofauna [97–99]. Peat carbon leaching into the wetland as DOC must in large part be assimilated by bacteria and thereby converted to particulate form for macroinvertebrates to access that peat carbon in their diet [100, 101]; we attempted to account for such processes in the TDFs (Table 2, Fig 3). Deposit-feeders with different feeding modes perhaps ingest different mixtures of fresh microalgae and bacteria. Moreover, we expect shifts in the relative production of microalgae and bacteria depending on the relative supply of inorganic nutrients and DOC in particular wetlands [12, 102].

Compared to other taxa, Physidae snails appeared to assimilate larger amounts of cyanobacteria relative to eukaryotic microalgae (Fig 3) [cf. 103–105]. This difference may partly reflect differential dependence of snails on surface biofilms relative to subsurface deposits [cf. 104]. It is unclear how the scraping of surfaces by snail radula might differ in effectiveness for feeding on surface biofilms or “mats” of cyanobacteria [43] relative to feeding on eukaryotic microalgae that may be less consolidated and more amenable to deposit-feeding.

A number of the δ^15^N values of invertebrates (Fig 3) initially appear too high for the taxa to be mainly consumers of the endmembers. Thus, some of the invertebrates might be predators, meaning that fractionation values for δ^15^N we used in mixing models are too low by the number of trophic levels [106]. The only published values we found for fractionation (Δ^15^N) between fresh microalgae and insect predators of herbivorous macroinvertebrates were 1.2 to 1.9‰ (S2 Table), which are not distinguishable from values for detritivores consuming bacteria or fungi (1.70 to 1.93‰, Table 2). Moreover, N in algal- or peat-derived DOM may be fractionated repeatedly in the microbial loop of the complex periphytic community by heterotrophic microbes (Table 2, S2 Table). Variable numbers of trophic transfers in the microbial loop can lead to increases in δ^15^N in macroinvertebrate consumers of the periphytic community that are comparable in magnitude to increases expected in predators of macroinvertebrates [69, 107, 108].

Coleoptera were mostly predatory Dytiscidae, but their δ^15^N values largely overlapped those of deposit-feeders presumed to be their prey. The only taxa in our wetlands other than Dytiscidae that could function as predators are Chironomidae in the subfamily Tanypodinae. However, Tanypodinae were a small fraction of Chironomidae in our study area, and are often only facultative predators (see section on Sample collection and processing in Methods). Thus, the relatively high numbers of chironomid samples with high δ^15^N (Fig 3) appear to represent mostly non-predatory taxa that were deposit-feeding in patches with exceptional turnover and fractionation of δ^15^N in the microbial loop. The high δ^15^N values of some grazing Physidae snails (Fig 3) also indicate that high δ^15^N values among our samples of detritivores resulted mostly from spatial patchiness of N recycling by the microbial loop in periphytic communities, rather than predation. Our results suggest that high δ^15^N in portions of chironomid communities in other studies may in fact not reflect carnivory [cf. 106].

Microalgae were clearly a dominant or major OM source in all wetland types except Deep Open Lakes where peat was dominant. Submersed macrophytes (*Arctophila*), which provide surfaces for epiphytic growth, are rare in Deep Open Lakes (as well as in Deep *Carex*). Inputs of labile DOM from thawing permafrost are generally greater in deeper lakes, because thawing sediments underneath deeper lakes extend below the typical active layer which supplies most DOM to shallow wetlands [57, 96]. This greater input of DOM may facilitate bacteria which are probably an intermediate step in invertebrate consumption of peat carbon. Deep Open Lakes were also the only wetland type in which Physidae snails did not include cyanobacteria as a major source of organic matter (Fig 3). Thus, there may be less growth of benthic microalgae and cyanobacteria in deeper, more light-limited waters.

### Relative biomasses of taxa among wetland types

Relative biomasses of invertebrate taxa varied substantially among wetland types. In cores (Fig 5), Chironomidae occurred consistently and often had the highest biomass, whereas Coleoptera ranged from having the highest biomass in Streams to being much less common or undetected in other wetland types. In net sweeps, the most striking overall pattern was that wetland types dominated by stands of macrophytes (*Arctophila* or *Carex*) had greater diversity and evenness among invertebrate taxa, as well as generally much higher total biomass of detritivores compared to Streams or Deep Open Lakes (Fig 6). These aspects emphasize the inordinate importance of shallow vegetated wetlands to the diversity and biomass of invertebrate prey available to avian consumers. Thus, although relative contributions of organic matter sources for different invertebrate taxa were largely consistent among wetland types, the greater invertebrate biomass and diversity in *Arctophila* and *Carex* wetlands, and their susceptibility to climate change [6], emphasize the importance of these wetland types in monitoring and conservation efforts [109].

### Climate-driven trajectories of wetland food webs

With climate warming, inputs to tundra wetlands of DOM leached from thawing permafrost will continue to increase [20, 62, 110]. Although the nutritional quality of organic matter in the active layer above permafrost is degraded over time with repeated thawing, leaching, and refreezing, the quality of organic matter within permafrost is well preserved [20]. Thus, DOM released from thawing permafrost tends to have a higher labile component which is more readily taken up by bacteria [111–113]. The resulting increase in bacterial activity likely explains the very high and increasing pCO_2_ levels in tundra wetlands near Utqiaġvik [22]. High pCO_2_ levels in turn facilitate microalgal production [114]. In the 1970s and 2010s, epibenthic algae in local wetlands were not nutrient-limited [19, 21, 86, 88], and recent permafrost thawing is also releasing abundant N and P into the wetlands [20]. Reduced exposure to light owing to burial by bioturbation appeared to be the main limitation of benthic algal biomass [32], but long Arctic photoperiods and extended ice-free durations will maintain high levels of incident light. With abundant supply of nutrients, light, and CO_2_, production of microalgae in these tundra wetlands is expected to be quite high (Fig 1; see also [10]).

Our results indicate that invertebrate food webs in these tundra wetlands depend strongly on direct herbivory of periphytic microalgae, and on ingestion of bacteria that consume microalgae or their exudates, vascular plant tissue or exudates, and peat DOM leached from surrounding tundra. Sources of organic matter to different invertebrate taxa in these tundra wetlands were also relatively constant across the six wetland types examined. Accordingly, we suggest that thawing of permafrost is unlikely to reduce or change these major organic matter sources or appreciably alter invertebrate diets. Thus, any climate-driven changes in invertebrate community structure may depend more on physiological responses or variations in life history relative to shifts in seasonal temperatures and phenology [115]. Indeed, macroinvertebrate assemblages in some deeper wetlands in this area showed little change between the 1970s and 2000s [11].

In our study area, shallow wetlands <1 ha declined by 30% in area and 17% in number over 65 years from 1948 to 2013 [6], a trend documented throughout circumpolar regions [5, 116, 117]. Declines in wetland area and number are accelerated by encroachment of emergent vegetation due to longer growing seasons [6, 118]. At the same time, increasing wetland water temperatures in summer [11] can lead to formation of taliks (belowground channels of thawed permafrost), resulting in rapid wetland drainage [117]. The latter effect may have already begun crossing a thawing threshold about 70 years before terrestrial permafrost thaw is predicted to become widespread in northern Alaska [119]. Shallow *Arctophila* and *Carex* wetlands, which have the highest invertebrate diversity and total biomass, are the wetlands most susceptible to these changes. Thus, climate-driven effects on overall invertebrate biomass or diversity available to avian consumers will likely depend not on shifts in OM sources, but more on reductions in overall number or area of these shallow emergent wetlands.

### Conservation implications for waterbirds

Tundra wetlands in the North American Arctic support a multitude of breeding waterbirds that migrate there from eastern Asia and throughout the western hemisphere [1–4]. These wetlands provide essential invertebrate foods for adults and young of a range of species, including sensitive populations of sea ducks (Mergini) and loons [120–122]. In our study area, sea ducks that wintered on the ocean relied heavily on nutrients from freshwater sources for egg production (89–95%), and either partly or entirely on freshwater nutrients for body maintenance during incubation (58–99%; [123]). For spectacled eiders (*Somateria fischeri*), duckling survival is correlated with duckling growth rates; and although predation may be the main proximate source of mortality, habitat conditions including food availability are likely the ultimate factor affecting duckling survival [124]. When northern pintail (*Anas acuta*) ducklings were introduced into individual tundra wetlands of the Yukon-Kuskokwim Delta of western Alaska, the ducklings quickly depleted invertebrate foods so that access to multiple alternative wetlands for feeding was critical (MWC Miller, pers observ).

Relative use of the different wetland types by sea ducks varies substantially [1, 125] (Miller et al. [Unpublished]). *Arctophila* and *Carex* wetlands have the highest invertebrate diversity and total biomass, and are by far the wetland types most used by breeding sea ducks in our study area. Despite lack of major shifts in organic matter sources and invertebrate assemblages within wetland types, the availability of shallow emergent wetlands as feeding areas for breeding birds may continue to decline. Reductions in these shallow wetland types has been concurrent with long-term decreases in breeding populations of king eiders (*Somateria spectabilis*), spectacled eiders, and long-tailed ducks (*Clangula hyemalis*) across the Arctic coastal plain [126]. Accordingly, documenting trends in the occurrence and types of Arctic tundra wetlands should be an important priority for conservation [6, 127].

## Supporting information

S1 Table Literature survey of stable isotope values for organic matter sources. These values were used as endmembers in mixing models of invertebrate diets in tundra wetlands near Utqiaġvik, Alaska.
(DOCX)

S2 Table Literature survey of trophic discrimination factors (TDFs) between consumers and their foods. These values were used in mixing models of invertebrate diets in tundra wetlands near Utqiaġvik, Alaska.
(DOCX)

S3 Table Carbon content per individual (including lipid) of invertebrate taxa in tundra wetlands near Utqiaġvik, Alaska, summer 2017 and 2018. (DOCX)

S4 Table Mean ± SE of stable isotope values (δ13C and δ15N) for each invertebrate taxon in different wetland types. N/A indicates no data for a given taxon. Numbers of wetlands of each wetland type that were sampled for each organism are in S7 and S8 Tables.
(DOCX)

S5 Table Diagnostics for MixSIAR Markov-chain Monte Carlo (MCMC) models. Gelman-Rubin values substantially greater than 1 indicate lack of model convergence. Gelman-Rubin diagnostics shown are number of parameters created by models, and the number (percentage in parentheses) exceeding thresholds of 1.01, 1.05, and 1.10. Geweke diagnostics are standard z-scores, so 5% of parameters in each chain are expected to exceed ± 1.96, with lower values indicating better model convergence. Geweke values shown in this table are the number of parameters exceeding ± 1.96 (percentage in parentheses).
(DOCX)

S6 Table Mean ± SE of mixing model estimates of the percentages of organic matter sources (%A = percentage algae, %PM = percentage peat and macrophytes) for different invertebrate taxa in different wetland types. (DOCX)

S7 Table Mean ± SE of biomasses in cores (mg C m^‒2^) of different invertebrate taxa in different tundra wetland types. Invertebrates were collected near Utqiaġvik, Alaska in summer 2017 and 2018. Means in the same row with the same superscript are not significantly different (PERMANOVA, *P* > 0.05). N/A indicates no data for a given taxon.
(DOCX)

S8 Table Mean ± SE of biomasses in net sweeps (mg C m^‒3^) of different invertebrate taxa in different tundra wetland types. Invertebrates were collected near Utqiaġvik, Alaska in summer 2017 and 2018. Means in the same row with the same superscript are not significantly different (PERMANOVA, *P* > 0.05). N/A indicates no data for a given taxon.
(DOCX)

## References

[1] Bergman RD, Howard RL, Abraham KF, Weller MW. Water birds and their wetland resources in relation to oil development at Storkersen Point, Alaska. Resource Publication 129. US Fish and Wildlife Service. 1977; 38 pp.

[2] Alerstam T, Gudmundsson GA. Bird orientation at high latitudes: flight routes between Siberia and North America across the Arctic Ocean. Proceedings of the Royal Society of London B. 1999; 266:2499–2505. doi: 10.1098/rspb.1999.0952 10693821 PMC1690484

[3] Bart J, Platte RM, Andres B, Brown S, Johnson JA, Larned W. Importance of the National Petroleum Reserve–Alaska for aquatic birds. Conservation Biology. 2013; 27:1304–1312. doi: 10.1111/cobi.12133 23937114

[4] Ward DH, Helmericks J, Hupp JW, McManus L, Budde M, Douglas DC, et al. Multi-decadal trends in spring arrival of avian migrants to the central Arctic coast of Alaska: effects of environmental and ecological factors. Journal of Avian Biology. 2016; 47:197–207.

[5] Smol JP, Douglas MSV. Crossing the final ecological threshold in high Arctic ponds. Proceedings of the National Academy of Sciences of the United States. 2007; 104:12395‒12397. doi: 10.1073/pnas.0702777104 17606917 PMC1941480

[6] Andresen CG, Lougheed VL Disappearing Arctic tundra ponds: fine-scale analysis of surface hydrology in drained thaw lake basins over a 65 year period (1948–2013). Journal of Geophysical Research Biogeosciences. 2015; 120:466‒479.

[7] Jorgenson MT, Shur YL, Pullman ER. Abrupt increase in permafrost degradation in Arctic Alaska. Geophysical Research Letters. 2006; 33:1–4.

[8] Vonk JE, Tank SE, Bowden WB, Laurion I, Vincent WF, Alekseychik P, et al. Reviews and syntheses: Effects of permafrost thaw on Arctic aquatic ecosystems. Biogeosciences. 2015; 12:7129–7167.

[9] Smol JP, Wolfe AP, Birks HJB, Douglas MSV, Jones VJ, Korhola A, et al. Climate-driven regime shifts in the biological communities of Arctic lakes. Proceedings of the National Academy of Sciences of the United States. 2005; 102:4397‒4402. doi: 10.1073/pnas.0500245102 15738395 PMC555516

[10] Chételat J, Cloutier L, Amyot M. Carbon sources for lake food webs in the Canadian High Arctic and other regions of Arctic North America. Polar Biology. 2010; 33:1111‒1123.

[11] Lougheed VL, Butler MG, McEwen DC, Hobbie JE. Changes in tundra pond limnology: re-sampling Alaskan ponds after 40 years. Ambio. 2011; 40:589–599. doi: 10.1007/s13280-011-0165-1 21954722 PMC3357870

[12] Mariash HL, Cazzanelli M, Rautio M, Hamerick L, Wooller MJ, Christoffersen KS. Changes in food web dynamics of low Arctic ponds with varying content of dissolved organic carbon. Arctic, Antarctic, and Alpine Research. 2018; 50:e1414472.

[13] Medina-Contreras D, Arenas-Gonzalez F, Cantera-Kintz J, Sanchez-Gonzalez A, Giraldo A. Food web structure and isotopic niche in a fringe macro-tidal mangrove system, tropical Eastern Pacific. Hydrobiologia. 2020; 847:3185–3199.

[14] Batzer DP, Sharitz RR. Ecology of freshwater and estuarine wetlands, 2^nd^ ed. Berkeley: University of California Press. 2014.

[15] Hart EA, Lovvorn JR. Algal vs. macrophyte inputs to food webs of inland saline wetlands. Ecology. 2003; 84:3317–3326.

[16] Trochine C, Villanueva VD, Brett MT. The ultimate peanut butter on crackers for *Hyalella*: diatoms on macrophytes rather than bacteria and fungi on conditioned terrestrial leaf litter. Freshwater Biology. 2021; 66:599–614.

[17] Findlay S, Carlough L, Crocker MT, Gill HK, Meyer JL, Smith PJ. Bacterial growth on macrophyte leachate and fate of bacterial production. Limnology and Oceanography. 1986; 31:1335‒1341.

[18] Sobczak WV. Epilithic bacterial responses to variations in algal biomass and labile dissolved organic carbon during biofilm colonization. Journal of the North American Benthological Society. 1996; 15:143‒154.

[19] Stanley DW. Productivity of epipelic algae in tundra ponds and a lake near Barrow, Alaska. Ecology. 1976; 57:1015–1024.

[20] Reyes FR, Lougheed VL. Rapid nutrient release from permafrost thaw in Arctic aquatic ecosystems. Arctic, Antarctic and Alpine Research. 2015; 47:35–48.

[21] Lougheed VL, Hernandez C, Andresen CG, Miller NA, Alexander V, Prentki R. 2015. Contrasting responses of phytoplankton and benthic algae to recent nutrient enrichment in Arctic tundra ponds. Freshwater Biology. 2015; 60:2169–2186.

[22] Lougheed VL, Tweedie CE, Andresen CG, Armendariz AM, Escarzaga SM, Tarin G. Patterns and drivers of carbon dioxide concentrations in aquatic ecosystems of the arctic coastal tundra. Global Biogeochemical Cycles. 2020; 34:1–13.

[23] Andresen CG, Lara MJ, Tweedie CE, Lougheed VL. Rising plant-mediated methane emissions from Arctic wetlands. Global Change Biology. 2017; 23:1128‒1139. doi: 10.1111/gcb.13469 27541438

[24] Dutta K, Schuur EAG, Neff JC, Zimov SA. Potential carbon release from permafrost soils of Northeastern Siberia. Global Change Biology. 2006; 12:2336–2351.

[25] Hugelius G, Routh J, Kuhry P, Crill P. Mapping the degree of decomposition and thaw remobilization potential of soil organic matter in discontinuous permafrost terrain. Journal of Geophysical Research. 2012; 117:G02030.

[26] Hugelius G, Strauss J, Zubrzycki S, Harden JW, Schuur EAG, Ping C-L, et al. Estimated stocks of circumpolar permafrost carbon with quantified uncertainty ranges and identified data gaps. Biogeosciences. 2014; 11:6573–6593.

[27] Brown J, Everett KR, Webber PJ, MacLean SF, Murray DF. The coastal tundra at Barrow. In Brown J, Miller PC, Tieszen LL, Bunnell FL, editors. An Arctic ecosystem: the coastal tundra at Barrow, Alaska. Stroudsburg, Dowden, Hutchinson & Ross. 1980. p. 1‒29.

[28] Hinkel KM, Eisner WR, Bockheim JG, Nelson FE, Peterson KM, Dai X. Spatial extent, age, and carbon stocks in drained thaw lake basins on the Barrow Peninsula, Alaska. Arctic, Antarctic, and Alpine Research. 2003; 35:291‒300.

[29] Lara MJ, McGuire AD, Euskirchen ES, Tweedie CE, Hinkel KM, Skurikhin AN, et al. Polygonal tundra geomorphological change in response to warming alters future CO_2_ and CH_4_ flux on the Barrow Peninsula. Global Change Biology. 2015; 21:1634–1651. 25258295 10.1111/gcb.12757

[30] Manley WF, Lestak LR, Tweedie C, Maslanik J. High-Resolution Quickbird Imagery and Related GIS Layers for Barrow, Alaska, USA, Version 1 [1 to 2 Aug 2002]. Boulder, Colorado USA. National Snow and Ice Data Center. 2006. Date Accessed 01-01-2014.

[31] Throckmorton HM, Newman BD, Heikoop JM, Perkins GB, Feng X, Graham DE, et al. Active layer hydrology in an Arctic tundra ecosystem: quantifying water sources and cycling using water stable isotopes. Hydrological Processes. 2016; 30:4972‒4986.

[32] Alexander V, Stanley DW, Daley RJ, McRoy CP. Primary producers. In Hobbie JE, editor. Limnology of tundra ponds, Barrow, Alaska. Stroudsburg: Dowden, Hutchinson & Ross; 1980. p. 179‒250.

[33] Haynes TB, Rosenberger AE, Lindberg MS, Whitman M, Schmutz JA. (2014) Patterns of lake occupancy by fish indicate different adaptations to life in a harsh Arctic environment. Freshwater Biology. 2014; 59:1884‒1896.

[34] Cowardin LM, Carter V, Golet FC, LaRoe ET. Classification of wetlands and deepwater habitats of the United States. FWS/OBS-79/31. US Fish and Wildlife Service, 1979; 131 pp.

[35] Derksen DV, Rothe TC, Eldridge WD. Use of wetland habitats by birds in the National Petroleum Reserve ‒ Alaska. Resource Publication 141, US Fish and Wildlife Service. 1981; 27 pp.

[36] Butler M, Miller MC, Mozley S. Macrobenthos. Hobbie JE, editor. Limnology of tundra ponds, Barrow, Alaska. Stroudsburg: Dowden, Hutchinson & Ross. 1980; p. 297‒339.

[37] Baker AS, McLachlan AJ. Food preferences of Tanypodinae larvae (Diptera: Chironomidae). Hydrobiologia. 1979; 62:283‒288.

[38] Sephton TW. Some observations on the food of larvae of *Procladius bellus* (Diptera: Chironomidae). Aquatic Insects. 1987; 9:195‒202.

[39] Butakka CMM, Ragonha FH, Train S, Pinha GD, Takeda AM. Chironomidae feeding habits in different habitats from a Neotropical floodplain: exploring patterns in aquatic food webs. Brazilian Journal of Biology. 2016; doi: 10.1590/1519-6984.14614 26909630

[40] Post DM, Layman CA, Arrington DA, Takimoto G, Quattrochi J, Montana CG. Getting to the fat of the matter: models, methods and assumptions for dealing with lipids in stable isotope analyses. Oecologia. 2007; 152:179‒189. doi: 10.1007/s00442-006-0630-x 17225157

[41] North CA, Lovvorn JR, Kolts JM, Cooper LW, Grebmeier JM. Discriminating trophic niches of carnivorous macroinvertebrates with gut contents, stable isotopes, and fatty acids. Marine Ecology Progress Series. 2019; 631:49‒66.

[42] O’Donnell JA, Carey MP, Koch JC, Xu X, Poulin BA, Walker J, et al. Permafrost hydrology drives the assimilation of old carbon by stream food webs in the Arctic. Ecosystems. 2020; 23:435‒453.

[43] Schell DM, Ziemann PJ. Natural carbon isotope tracers in arctic aquatic food webs. In Rundel PW, Ehleringer JR, Nagy KA, editors. Stable isotopes in ecological research. New York: Springer-Verlag. 1989; p. 230‒251.

[44] Peterson BJ, Fry B, Deegan L, Hershey A. The trophic significance of epilithic algal production in a fertilized tundra river ecosystem. Limnology and Oceanography. 1993; 38:872‒878.

[45] Skrzypek G, Paul D, Wotjuń B. Stable isotope composition of plants and peat from Arctic mire and geothermal area in Iceland. Polish Polar Research. 2008; 29:365–376.7

[46] Gu B, Alexander V. Estimation of N_2_ fixation based on differences in the natural abundance of ^15^N among freshwater N_2_-fixing and non-N_2_-fixing algae. Oecologia. 1993; 96:43–48. 28313752 10.1007/BF00318029

[47] Evans SL, Anderson WT, Jochem FJ. Spatial variability in Florida Bay particulate organic matter composition: combining flow cytometry with stable isotope analyses. Hydrobiologia. 2006; 569:151–165.

[48] Fischer H. The role of biofilms in the uptake and transformation of dissolved organic matter. In Findlay SEG, Sinsabaugh RL, editors. Aquatic ecosystems: interactivity of dissolved organic matter. New York: Academic Press; 2003. p. 285‒313.

[49] Ishikawa NF, Yamane M, Suga H, Ogawa NO, Yokoyama Y, Ohkouchi N. Chlorophyll *a*-specific Δ^14^C, δ^13^C and δ^15^N values in stream periphyton: implications for aquatic food web studies. Biogeosciences. 2015; 12:6781‒6789.

[50] Frost PC, Hillebrand H, Kahlert M. Low algal carbon content and its effect on the C:P stoichiometry of periphyton. Freshwater Biology. 2005; 50:1800‒1807.

[51] Kivila EH, Luoto TP, Rantala MV, Kiljunen M, Rautio M, Nevalainen L. Environmental controls on benthic food web functions and carbon use in subarctic lakes. Freshwater Biology. 2019; 64:643‒658.

[52] Post DM. Using stable isotopes to estimate trophic position: models, methods, and assumptions. Ecology. 2002; 83:703‒718.

[53] Grippo MA, Fleeger JW, Dubois SF, Condrey R. Spatial variation in basal resources supporting benthic food webs revealed for the inner continental shelf. Limnology and Oceanography. 2011; 56(3):841‒856.

[54] Throckmorton HM, Heikoop JM, Newman BD, Altmann GL, Conrad MS, Muss JD, et al. Pathways and transformations of dissolved methane and dissolved inorganic carbon in Arctic tundra watersheds: evidence from analysis of stable isotopes. Global Biogeochemical Cycles. 2015; 29:1893‒1910.

[55] Quinton WL, Gray DM, Marsh P. Subsurface drainage from hummock-covered hillslopes in the Arctic tundra. Journal of Hydrology. 2000; 237:113‒125.

[56] Quinton WL, Hayashi M, Carey SK. Peat hydraulic conductivity in cold regions and its relation to pore size and geometry. Hydrological Processes. 2008; 22:2829‒2837.

[57] Dean JF, Meisel OH, Rosco MM, Marchesini LB, Garnett MH, Lenderink H, et al. East Siberian Arctic inland waters emit mostly contemporary carbon. Nature Communications. 2020; 11:1627. doi: 10.1038/s41467-020-15511-6 32242076 PMC7118085

[58] Ingram HAP. Soil layers in mires: function and terminology. Journal of Soil Science. 1978; 29:224‒227.

[59] Szajdak LW, Lapshina ED, Gaca W, Styla K, Meysner T, Szczepanski M, et al. Physical, chemical and biochemical properties of western Siberia *Sphagnum* and *Carex* peat soils. Environmental Dynamics and Global Climate Change. 2016; 2:13‒25.

[60] Michaelis D, Mrotzek A., Couwenberg J Roots, tissues, cells and fragments ‒ how to characterize peat from drained and rewetted fens. Soil Systems. 2020; 4:12.

[61] Jones RI, Grey J. Biogenic methane in freshwater food webs. Freshwater Biology 2011; 56:213‒229.

[62] Drake TW, Wickland KP, Spencer RGM, McKnight DM, Striegl RG. Ancient low-molecular-weight organic acids in permafrost fuel rapid carbon dioxide production upon thaw. Proceedings of the National Academy of Sciences of the United States. 2015; 112:13946‒13951. doi: 10.1073/pnas.1511705112 26504243 PMC4653224

[63] Laurion I, Miladenov N. Dissolved organic matter photolysis in Canadian Arctic thaw ponds. Environmental Research Letters. 2013; 8:035026.

[64] Fellman JB, Hood E, Raymond PA, Hudson J, Bozeman M, Arimitsu M. Evidence for the assimilation of ancient glacier organic carbon in a proglacial stream food web. Limnology and Oceanography. 2015; 60:1118‒1128.

[65] Mann CJ, Wetzel RG. Loading and utilization of dissolved organic carbon from emergent macrophytes. Aquatic Botany. 1996; 53:61‒72.

[66] Gutierrez-Rodriguez A, Decima M, Popp BN, Landry MR. Isotopic invisibility of protozoan trophic steps in marine food webs. Limnology and Oceanography. 2014; 59:1590‒1598.

[67] Park JY, Jung J-H, Kwak JH, Park HG, Kang C-K, Park HJ. Trophic enrichment factors of carbon and nitrogen isotopic ratios (del^13^C and del^15^N) in four marine ciliates. Frontiers in Microbiology. 2021; 12: article 721157. 10.3389/fmicb.2021.721157 PMC8495318 34630351

[68] Wetzel RG. Dissolved organic carbon: detrital energetics, metabolic regulators, and drivers of ecosystem stability of aquatic ecosystems. In Findlay SEG, Sinsabaugh RL, editors. Aquatic ecosystems: interactivity of dissolved organic matter. New York: Academic Press. 2003; pp. 455‒477.

[69] Steffan SA, Chikaraishi Y, Dharampal PS, Pauli JN, Guedot C, Ohkouchi N. Unpacking brown food-webs: animal trophic identity reflects rampant microbivory. Ecology and Evolution. 2017; 7:3532‒3541. doi: 10.1002/ece3.2951 28515888 PMC5433990

[70] Müller-Navarra DC, Brett MT, Liston AM, Goldman CR. A highly unsaturated fatty acid predicts carbon transfer between primary producers and consumers. Nature. 2000; 403:74–77. doi: 10.1038/47469 10638754

[71] Larsen T, Pollierer MM, Holmstrup M, D’Annibale A, Maraldo K, Andersen N, et al. Substantial nutritional contribution of bacterial amino acids to earthworms and enchytraeids: A case study from organic grasslands. Soil Biology and Biochemistry. 2016; 99:21–27.

[72] Moran MA, Hodson RE. Bacterial secondary production on vascular plant detritus: relationships to detritus composition and degradation rate. Applied Environmental Microbiology. 1989; 55:2178–2189. doi: 10.1128/aem.55.9.2178-2189.1989 2802603 PMC203053

[73] Kuehn KA, Lemke MJ, Suberkropp K, Wetzel RG. Microbial biomass and production associated with decaying leaf litter of the emergent macrophyte *Juncus effusus*. Limnology and Oceanography. 2000; 45:862‒870.

[74] Hieber M, Gessner MO. Contribution of stream detritivores, fungi, and bacteria to leaf breakdown based on biomass estimates. Ecology. 2002; 83:1026‒1038.

[75] Hobbie JE, Traaen T, Rublee P, Reed JP, Miller MC, Fenchel T. Decomposers, bacteria, and microbenthos. In Hobbie JE, editor. Limnology of tundra ponds, Barrow Alaska. Stroudsburg: Dowden, Hutchinson & Ross. 1980. p. 340‒387.

[76] Stock BC, Semmens BX. MixSIAR GUI user manual. Version 3.1. 2016. https://github.com/brianstock/MixSIAR. doi: 10.5281/zenodo.1209993

[77] Anderson MJ, Gorley RN, Clarke KR. PERMANOVA+ PRIMER: Guide to software and statistical methods. Plymouth, UK: PRIMER-E; 2008.

[78] Clarke KR, Gorley RN. PRIMER v7: User manual/tutorial. Plymouth, UK: PRIMER-E. 2015.

[79] Fry B. Alternative approaches for solving underdetermined isotope mixing problems. Marine Ecology Progress Series. 2013; 472:1‒13.

[80] Grey J. The incredible lightness of being methane-fuelled: stable isotopes reveal alternative energy pathways in aquatic ecosystems and beyond. Frontiers in Ecology and Evolution. 2016; 4: Article 6.

[81] Yasuno N, Shikano S, Shimada T, Shindo K, Kikuchi E. Comparison of the exploitation of methane-derived carbon by tubicolous and non-tubicolous chironomid larvae in a temperate eutrophic lake. Limnology. 2013; 14:239‒246.

[82] Agasild H, Zingel P, Tuvikene L, Tuvikene A, Timm H, Feldmann T, et al. Biogenic methane contributes to the food web of a large, shallow lake. Freshwater Biology, 2014; 59:272‒285.

[83] Knoblauch C, Spott O, Evgrafova S, Kutzbach L, Pfeiffer E-M. Regulation of methane production, oxidation, and emission by vascular plants and bryophytes in ponds of the northeast Siberian polygonal tundra. Journal of Geophysical Research: Biogeosciences. 2015; 120:2525‒2541.

[84] Jones RI, Carter CE, Kelly A, Ward S, Kelly DJ, Grey J. Widespread contribution of methane-cycle bacteria to the diets of lake profundal chironomid larvae. Ecology. 2008; 89:857‒864. doi: 10.1890/06-2010.1 18459348

[85] Prentki RT, Miller MC, Barsdate RJ, Alexander V, Kelley J, Coyne P. Chemistry. In Hobbie, editor. Limnology of tundra ponds, Barrow Alaska. Stroudsburg: Dowden, Hutchinson & Ross. 1980; p. 76‒178.

[86] Lennon JT, Faiia AM, Feng X, Cottingham KL. Relative importance of CO_2_ recycling and CH_4_ pathways in lake food webs along a dissolved organic carbon gradient. Limnology and Oceanography. 2006; 51:1602‒1613.

[87] Stanley DW. A carbon flow model of epipelic algal productivity in Alaskan tundra ponds. Ecology. 1976; 57:1034–1042.

[88] Stanley DW, Daley RJ. Environmental control of primary productivity in Alaskan tundra ponds. Ecology. 1976; 57:1025–1033.

[89] Ward CP, Nalven SG, Crump BC, Kling GW, Cory RM. Photochemical alteration of organic carbon draining permafrost soils shifts microbial metabolic pathways and stimulates respiration. Nature Communications. 2017; 8:1–8. 10.1038/s41467-017-00759-2 PMC5626735 28974688

[90] Ishikawa NF, Doi H, Finlay JC. Global meta-analysis for controlling factors on carbon stable isotope ratios of lotic periphyton. Oecologia. 2012; 170:541‒549. doi: 10.1007/s00442-012-2308-x 22466861

[91] Badger M. The roles of carbonic anhydrases on photosynthetic CO_2_ concentrating mechanisms. Photosynthesis Research. 2003; 77:83‒94. 16228367 10.1023/A:1025821717773

[92] Merz-Preiβ M, Riding R. Cyanobacterial tufa calcification in two freshwater streams: ambient environment, chemical thresholds and biological processes. Sedimentary Geology. 1999; 126:103‒124.

[93] Benavides M, Berthelot H, Duhamel S, Raimbault P, Bonnet S. Dissolved organic matter uptake by Trichodesmium in the southwest Pacific. Scientific Reports. 2017; 7:41315. doi: 10.1038/srep41315 28117432 PMC5259775

[94] Muñoz-Marin MC, Gomez-Baena G, Lopez-Lozano A, Moreno-Cabezuelo JA, Diez J, Garcia-Fernandez JM. Mixotrophy in marine picocyanobacteria: use of organic compounds by *Prochlorococcus* and *Synechococcus*. ISME Journal. 2020; 14:1065–1073. 32034281 10.1038/s41396-020-0603-9 PMC7174365

[95] Feng X, Bandyopadhyay A, Berla B, Page L, Wu B, Pakrasi HB, et al. Mixotrophic and photoheterotrophic metabolism in *Cyanotheca* sp. ATCC 51142 under continuous light. Microbiology. 2010; 156:2566–2574. 20430816 10.1099/mic.0.038232-0

[96] Barnes RT, Butman DE, Wilson HF, Raymond PA. Riverine export of aged carbon driven by flow path depth and residence time. Environmental Science and Technology. 2018; 52:1028‒1035. doi: 10.1021/acs.est.7b04717 29313674

[97] Fenchel T. The quantitative importance of the benthic microfauna of an Arctic tundra pond. Hydrobiologia. 1975; 46:45‒464.

[98] Decho AW, Castenholz RW. Spatial patterns and feeding of meiobenthic harpacticoid copepods in relation to resident microbial flora. Hydrobiologia. 1986; 131:87‒96.

[99] North CA, Lovvorn JR, Kolts JM, Brooks ML, Cooper LW, Grebmeier JM. Deposit-feeder diets in the Bering Sea: potential effects of climatic loss of sea ice-related microalgal blooms. Ecological Applications. 2014; 24:1525‒1542. 29160671

[100] Alber M, Valiela I. Production of microbial organic aggregates from macrophyte-derived dissolved organic matter. Limnology and Oceanography. 1994; 39:37‒50.

[101] Alber M, Valiela I. Organic aggregates in detrital food webs: incorporation by bay scallops *Argopecten irradians*. Marine Ecology Progress Series. 1995; 121:117‒124.

[102] Myers JM, Kuehn KA, Wyatt KH. Carbon subsidies shift a northern peatland biofilm community towards heterotrophy in low but not in high nutrient conditions. Freshwater Biology. 2021; 66:589‒598.

[103] Lance E, Brient L, Bormans M, Gerard C. Interactions between cyanobacteria and gastropods I. Ingestion of toxic *Planktothrix agardhii* by *Lymnaea stagnalis* and the kinetics of microcystin bioaccumulation and detoxification. Aquatic Toxicology. 2006; 79:140–148. 16837077 10.1016/j.aquatox.2006.06.004

[104] Karlson AML, Gorokhova E, Elmgren R. Nitrogen fixed by cyanobacteria is utilized by deposit-feeders. PLoS One. 2014; 9:e104460. doi: 10.1371/journal.pone.0104460 25105967 PMC4126700

[105] Groendahl S, Fink P. High dietary quality of non-toxic cyanobacteria for a benthic grazer and its implications for the control of cyanobacterial biofilms. BMC Ecology. 2017; 17:20, doi: 10.1186/s12898-017-0130-3 28521755 PMC5437396

[106] Reuss NS, Hamerlik L, Velle G, Michelson A, Pedersen O, Brodersen KP. Stable isotopes reveal that chironomids occupy several trophic levels within West Greenland lakes: implications for food web studies. Limnology and Oceanography. 2013; 58:1023‒1034.

[107] McGoldrick DJ, Barton DR, Power M, Scott RW, Butler BJ. Dynamics of bacteria-substrate stable isotope separation: dependence on substrate availability and implications for aquatic food web studies. Canadian Journal of Fisheries and Aquatic Sciences. 2008; 65:1983‒1990.

[108] Steffan SA, Chikaraishi Y, Currie CR, Horn H, Gaines-Day HR, Pauli JN, et al. Microbes are trophic analogs of animals. Proceedings of National Academy of Sciences. 2015; 112:15119‒15124. doi: 10.1073/pnas.1508782112 26598691 PMC4679051

[109] Wauchope HS, Shaw JD, Varpe O, Lappo EG, Boertmann D, Lanctot RB, et al. Rapid climate-driven loss of breeding habitat for Arctic migratory birds. Global Change Biology. 2017; 23:1085‒1094. doi: 10.1111/gcb.13404 27362976

[110] Wauthy M, Rautio M, Christoffersen KS, Forsstrom L, Laurion I, Mariash HL, et al. Increasing dominance of terrigenous organic matter in circumpolar freshwaters due to permafrost thaw. Limnology and Oceanography Letters. 2018; 3:186‒198.

[111] Abbott BW, Larouche JR, Jones JB, Bowden WB, Balser AW. Elevated dissolved organic carbon biodegradability from thawing and collapsing permafrost. Journal of Geophysical Research: Biogeosciences. 2014; 119:2049‒2063.

[112] Ward CP, Cory RM. Chemical composition of dissolved organic matter draining permafrost soils. Geochimica et Cosmochimica Acta. 2015; 167:63‒79.

[113] Selvam BP, Lapierre J-F, Guillemette F, Voigt C, Lamprecht RE, Biasi C, et al. Degradation potentials of dissolved organic carbon (DOC) from thawed permafrost peat. Scientific Reports. 2017; 7:45811. doi: 10.1038/srep45811 28378792 PMC5395014

[114] Brown T-RW, Lajeunesse MJ, Scott KM. Strong effects of elevated CO_2_ on freshwater microalgae and ecosystem chemistry. Limnology and Oceanography. 2020; 65:304‒313.

[115] Klobucar SL, Gaeta JW, Budy P. A changing menu in a changing climate: using experimental and long-term data to predict invertebrate prey biomass and availability in lakes of Arctic Alaska. Freshwater Biology. 2018; 63:1352‒1364.

[116] Smith LC, Sheng Y, MacDonald GG, Hinzman LD. Disappearing Arctic lakes. Science. 2005; 308:1429. doi: 10.1126/science.1108142 15933192

[117] Marsh P, Russell M, Pohl S, Haywood H, Onclin C. Changes in thaw lake drainage in the western Canadian Arctic from 1950‒2000. Hydrological Processes. 2009; 23:145‒158.

[118] Villarreal S, Hollister RD, Johnson DR, Lara MJ, Webber PJ, Tweedie CE. Tundra vegetation change near Barrow, Alaska (1972–2010). Environmental Research Letters. 2012; 7. doi: 10.1088/1748-9326/7/1/015508

[119] Arp CD, Jones BM, Grosse G, Bondurant AC, Romanovsky VE, Hinkel KM, et al. Threshold sensitivity of shallow Arctic lakes and sublake permafrost to changing winter climate. Geophysical Research Letters. 2016; 42:6358‒6365.

[120] Kondratyev AV. Foraging strategies and habitat use of sea ducks breeding in northeast Russia. In Goudie RI, Petersen MR, Robertson GJ, editors. Behaviour and ecology of sea ducks. Canadian Wildlife Service Occasional Papers. 1999; 100:52‒59.

[121] Howard RL, Kertell K, Truett JC. 2000. Freshwater invertebrates: their regulation and importance to vertebrates. In Truett JC, Johnson SR, editors. The natural history of an oilfield. San Diego: Academic Press. 2000; p. 307‒326.

[122] Rizzolo DJ. Contrasting diet, growth, and energy provisioning in loons breeding sympatrically in the Arctic. PhD thesis. Fairbanks: University of Alaska. 2017.

[123] Miller MWC, Lovvorn JR, Graff N, Stellrecht N. Use of marine vs. freshwater nutrients for egg-laying and incubation by sea ducks breeding in Arctic tundra. Ecosphere. 2022; 12: Forthcoming.

[124] Flint PL, Morse JA, Grand JB, Moran CL. Correlated growth and survival of juvenile spectacled eiders: evidence of habitat limitation? Condor. 2006; 108:901–911.

[125] Derksen DV, Rothe TC, Eldridge WD. Use of wetland habitats by birds in the National Petroleum Reserve–Alaska. Resource Publication 141. US Fish and Wildlife Service. 1981.

[126] Amundson C, Flint P, Stehn R, Platte R, Wilson H, Larned W, et al. Spatio-temporal population change of Arctic-breeding waterbirds on the Arctic Coastal Plain of Alaska. Avian Conservation and Ecology. 2019; 14, 18 pp.

[127] Muster S, Heim B, Abnizova A, Boike J. Water body distributions across scales: a remote sensing based comparison of three arctic tundra wetlands. Remote Sensing. 2013; 5:1498–1523.

