# Supplementary material for: Organic matter sources and flows in tundra wetland food webs
Source: PLoS One. 2023 May 26;18(5):e0286368. doi: 10.1371/journal.pone.0286368 (PMC10218757; doi:10.1371/journal.pone.0286368)
Supplement: S1 Table — These values were used as endmembers in mixing models of invertebrate diets in tundra wetlands near Utqiaġvik, Alaska. (DOCX) [file pone.0286368.s001.docx]

**S1 Table. Literature survey of stable isotope values for organic matter sources.** These values were used as endmembers in mixing models of invertebrate diets in tundra wetlands near Utqiaġvik, Alaska.

| **Endmember** | **δ^13^C**  **(‰)** | **δ^15^N**  **(‰)** | **Reference** | **Location** | **Environment** | **Sample type** |
| --- | --- | --- | --- | --- | --- | --- |
| Algae | -40.5 |  | O'Donnell et al. (2020) | NW Alaska | 4 streams in ice-rich permafrost: | Biofilm scraped |
| Algae | -38.8 |  |  |  | Arctic tundra, boreal spruce | from rocks |
| Algae | -29.5 |  |  |  |  |  |
| Algae | -38.2 |  |  |  |  |  |
| Algae | -36.75 |  | Grand mean |  |  |  |
| Algae | 4.93 |  | SD (*n* = 4) |  |  |  |
| Algae | 2.47 |  | SE (*n* = 4) |  |  |  |
|  |  |  |  |  |  |  |
| Peat | -28.65 |  | Schell & Ziemann (1989) | Kuparuk River, AK | River, near Prudhoe Bay, ACP |  |
| Peat | -28.34 |  |  | Kuparuk River, AK | River, Near Tulik Lake, ACP |  |
| Peat | -28.5 |  |  | Sagavanirktok R., AK | River, Arctic Coastal Plain |  |
| Peat | -27.6 |  |  | Streambank, AK | Streambank, Arctic Coastal Plain |  |
| Peat | -27.6 |  |  | Pond shoreline, AK | Pond, ACP, <22 cm & 23-41 cm depth | |
| Peat | -26.80 | 1.1 | Peterson et al. (1993) | Kuparuk River, AK | River, Arctic Coastal Plain |  |
| Peat | -27.98 | -1.41 | Skrzypek et al. (2008) | Iceland | Arctic fen | *Carex* peat |
| Peat | -29.19 | -1.0 |  |  |  |  |
| Peat | -27.95 | -1.26 |  |  |  |  |
| Peat | -28.18 | -1.02 |  |  |  |  |
| Peat | -29.16 | -0.94 |  |  |  |  |
| Peat | -28.18 | -0.76 | Grand mean |  |  |  |
| Peat | 0.71 | 0.93 | SD (*n* = 11, 6) |  |  |  |
| Peat | 0.21 | 0.38 | SE (*n* = 11, 6) |  |  |  |
|  |  |  |  |  |  |  |
| Cyanobacteria | -20.2 |  | Schell & Ziemann (1989) | Near Prudhoe Bay, AK | Pond, Arctic Coastal Plain | *Nostoc* mats |
| Cyanobacteria |  | 0.36 | Gu & Alexander (1993) | Arctic & subarctic, AK | Ponds at 4 sites, including ACP | 7 taxa |
| Cyanobacteria |  | -0.27 |  |  |  |  |
| Cyanobacteria |  | 0.36 |  |  |  |  |
| Cyanobacteria |  | 3.45 |  |  |  |  |
| Cyanobacteria |  | 0.04 |  |  |  |  |
| Cyanobacteria |  | 2.27 |  |  |  |  |
| Cyanobacteria |  | 0.41 |  |  |  |  |
| Cyanobacteria | -25.3 | 2.25 | Evans et al. (2006) | South Florida | FW discharge into marine bay | *Synechococcus* |
| Cyanobacteria | -22.3 | 2.88 |  |  |  |  |
| Cyanobacteria | -22.60 | 1.31 | Grand mean |  |  |  |
| Cyanobacteria | 2.56 | 1.40 | SD (*n* = 3, 9) |  |  |  |
| Cyanobacteria | 1.48 | 0.47 | SE (*n* = 3, 9) |  |  |  |

**________________________________________________________________________________________________________________________**

Evans SL, Anderson WT, Jochem FJ. 2006. Spatial variability in Florida Bay particulate organic matter composition: combining flow cytometry with stable isotope analyses. Hydrobiologia 569:151–165.

Gu B, Alexander V. 1993. Estimation of N_2_ fixation based on differences in the natural abundance of ^15^N among freshwater N_2_-fixing and non-N_2_-fixing algae. Oecologia 96:43–48.

O'Donnell JA, Carey MP, Koch JC, Xu X, Poulin BA, Walker J, Zimmerman CE. 2020. Permafrost hydrology drives the assimilation of old carbon by stream food webs in the Arctic. Ecosystems 23:435‒453.

Peterson BJ, Fry B, Deegan L, Hershey A. 1993. The trophic significance of epilithic algal production in a fertilized tundra river ecosystem. Limnology and Oceanography 38:872‒878.

Schell DM, Ziemann PJ. 1989. Natural carbon isotope tracers in arctic aquatic food webs. Rundel PW, Ehleringer JR, Nagy KA, editors. Stable isotopes in ecological research. New York, Springer-Verlag. p 230‒251.

Skrzypek G, Paul D, Wotjuń B. 2008. Stable isotope composition of plants and peat from Arctic mire and geothermal area in Iceland. Polish Polar Research 29:365–376.
