# Supplementary material for: Organic matter sources and flows in tundra wetland food webs
Source: PLoS One. 2023 May 26;18(5):e0286368. doi: 10.1371/journal.pone.0286368 (PMC10218757; doi:10.1371/journal.pone.0286368)
Supplement: S2 Table — These values were used in mixing models of invertebrate diets in tundra wetlands near Utqiaġvik, Alaska. (DOCX) [file pone.0286368.s002.docx]

**S2 Table.** **Literature survey of trophic discrimination factors (TDFs) between consumers and their foods**. These values were used in mixing models of invertebrate diets in tundra wetlands near Utqiaġvik, Alaska.

_____________________________________________________________________________________

**Δδ^13^C Δδ^15^N**

**Trophic transfer (‰) (‰) Reference**

**_____________________________________________________________________________________________**

1. **Fresh algal cells to invertebrate herbivores**

Fresh algal cells to invertebrate herbivores 0.6 (1.4)* Bunn et al. (2013)

Lettuce leaves to terrestrial snail (*Helix aspersa*) 1.10 ‒0.36 Caut et al. (2009, Table A1)

Deposited seston to two small marine snails 0.38 0.01 Caut et al. (2009, Table A1)

Fresh macroalgae (*Ulva, Gelidium*) to 2 amphipods ‒1.20 0.90 Caut et al. (2009, Table A1)

Fresh macroalgae (brown) to 3 marine snails 1.44 Chikaraishi et al. (2007)

**Mean 0.09 0.66**

1. **Fresh algal cells to predators of invert herbivores** 1.8 (4.1)* Bunn et al. (2013)

Mean **1.0** **3.0** Assumed value (no data found)

1. **Cyanobacteria to invertebrate consumers**

*Spirulina* to chironomid larvae **0.3 1.1** Caut et al. (2009, Table A1)

1. **Fresh algae or non-living OM to bacteria and protists**

Glucose medium to bacteria ‒0.6 Blair et al. (1985)

Terrestrial soil organic carbon to bacteria 2.0 Santruckova et al. (2000)

A diversity of substrates to bacteria 1.3 Pelz et al. (1998)

Fresh algal cells to ciliates 0.6 1.2 Park et al. (2021)

Fresh algal cells to dinoflagellate (protist) 0 Gutierrez-Rodriguez et al. (2014)

**Mean 0.82 0.6**

1. **Fresh algae or detritus to bacteria & protists to invert detritivores**

From items 3 and 4 above, algae to microbes to

inverts: Δ^13^C = 0.82 + 0.3, Δ^15^N = 0.6 + 1.1 **1.12 1.7**

1. **Peat tissue to DOM to bacteria & protists to invert detritivores**

Fractionation between soil OM and water-soluble

OM over 1‒90 d (DOM derived from peat via

bacterial decomposition) 0.37 ‒0.89 Lerch et al. (2011)

Fractionation between water-soluble OM (DOM)

to microbial biomass (cells and exopolymer)

over 30‒90 d (allows delay for leaching into pond) 0.35 3.17 Lerch et al. (2011)

Sum from peat tissue to DOM to pond bacteria to

invert detritivore:

Δ^13^C = 0.37 + 0.35 + 0.3, Δ^15^N = ‒0.89 + 3.17 + 1.1 **1.02 3.38**

1. **Macrophyte tissue to invert detritivores via fungi**

Plant-based growth medium to 5 fungal taxa ‒0.55 ‒1.27 Semenina & Tiunov (2010)

Macrophyte litter (alder leaves) to fungi ‒0.8 0.34 Constantini et al. (2014, Fig. 3)

Mean ‒0.68 ‒0.46

Fungi on growth medium to Collembola 1.0 1.4 Potapov et al. (2013, p 1069)

Calculated macrophyte litter to fungi to invert

detritivore:

Δ^13^C = ‒0.68 + 1.0, Δ^15^N = ‒0.46 + 1.4 0.32 0.94

Leaf litter (aspen or maize) to fungi to Collembola 3.6 1.5 Potapov et al. (2013, p 1069)

Aged aquatic macrophyte litter to chironomid larvae ‒4.7 5.0 McGoldrick et al. (2008)

*Phragmites* litter to two salt marsh snails 4.20 ‒0.71 Caut et al. (2009, Table A1)

**Mean 1.03 1.93**

1. **Deposit-feeders to invertebrate predators**

Invertebrate herbivores to invertebrate predators 1.2 (1.9)* Bunn et al. (2013)

Mean **1.6**

**_____________________________________________________________________________________________**

* For Bunn et al. (2013), the first value is from sites in Australia and New Guinea and the second value is from literature review.

Blair N, Leu A, Munoz E, Olsen J, Kwong E, Des Marais D. 1985. Carbon isotopic fractionation in heterotrophic microbial metabolism. Applied and Environmental Microbiology 50:996‒1001.

Bunn SE, Leigh C, Jardine TD. 2013. Diet-tissue fractionation of del^15^N by consumers from streams and rivers. Limnology and Oceanography 58:765‒773.

Butler M, Miller MC, Mozley S. 1980. Macrobenthos. In Hobbie JE (ed.) Limnology of tundra ponds, Barrow, Alaska. Dowden, Hutchinson & Ross, Stroudsburg, PA. p 297‒339.

Caut S, Angulo E, Courchamp F. 2009. Variation in discrimination factors (Δ^15^N and Δ^13^C): the effect of diet isotopic values and applications for diet reconstruction. Journal of Applied Ecology 46:443‒453.

Constantini ML, Calizza E, Rossi L. 2014. Stable isotope variation during fungal colonisation of leaf detritus in aquatic environments. Fungal Ecology 11:154‒163.

Dijkstra P, Ishizu A, Doucett R, Hart SC, Schwartz E, Menyailo OV, Hungate BA. 2006. ^13^C and ^15^N natural abundance of the soil microbial biomass. Soil Biology & Biogeochemistry 38:3257‒3266.

Gutierrez-Rodriguez A, Decima M, Popp BN, Landry MR. 2014. Isotopic invisibility of protozoan trophic steps in marine food webs. Limnology and Oceanography 59:1590‒1598.

Hieber M, Gessner MO. 2002. Contribution of stream detritivores, fungi, and bacteria to leaf breakdown based on biomass estimates. Ecology 83:1026‒1038.

Hobbie JE, Traaen T, Rublee P, Reed JP, Miller MC, Fenchel T. 1980. Decomposers, bacteria, and microbenthos. In Hobbie JE, ed. Limnology of tundra ponds, Barrow, Alaska. Dowden, Hutchinson & Ross, Stroudsburg, PA. p 340‒387.

Kuehn KA, Lemke MJ, Suberkropp K, Wetzel RG. 2000. Microbial biomass and production associated with decaying leaf litter of the emergent macrophyte *Juncus effusus*. Limnology and Oceanography 45:862‒870.

Lerch TZ, Nunan N, Dignac M-F, Chenu C, Mariotti A. 2011. Variations in microbial isotopic fractionation during soil organic matter decomposition. Biogeochemistry 106:5‒21.

Lougheed VL, Butler MG, McEwen DC, Hobbie JE. 2011. Changes in tundra pond limnology: re-sampling Alaskan ponds after 40 years. AMBIO 40:589‒599.

McGoldrick DJ, Barton DR, Power M, Scott RW, Butler BJ. 2008. Dynamics of bacteria-substrate stable isotope separation: dependence on substrate availability and implications for aquatic food web studies. Canadian Journal of Fisheries and Aquatic Sciences 65:1983‒1990.

Park JY, Jung J-H, Kwak JH, Park HG, Kang C-K, Park HJ. 2021. Trophic enrichment factors of carbon and nitrogen isotopic ratios (del^13^C and del^15^N) in four marine ciliates. Frontiers in Microbiology 12:article 721157.

Pelz O, Cifuentes LA, Hammer BT, Kelley CA, Coffin RB. 1998. Tracing the assimilation of organic compounds using δ^13^C analysis of unique amino acids in the bacterial peptidoglycan cell wall. FEMS Microbiology Ecology 25:229‒240.

Potapov AM, Semenina EF, Kurakov AV, Tiunov AV. 2013. Large ^13^C/^12^C and small ^15^N/^14^N isotope fractionation in an experimental detrital foodweb (litter-fungi-collembolans). Ecological Research 28:1069‒1079.

Santruckova H, Bird MI, Lloyd J. 2000. Microbial processes and carbon-isotope fractionation in tropical and temperate grassland soils. Functional Ecology 14:108‒114.

Semenina EE, Tiunov AV. 2010. Isotopic fractionation by saprotrophic microfungi: effects of species, temperature and the age of colonies. Pedobiologia 53:213‒217.
