# Supplementary material for: Organic matter sources and flows in tundra wetland food webs
Source: PLoS One. 2023 May 26;18(5):e0286368. doi: 10.1371/journal.pone.0286368 (PMC10218757; doi:10.1371/journal.pone.0286368)
Supplement: S3 Table — (DOCX) [file pone.0286368.s003.docx]

**S3 Table.** **Carbon content per individual (including lipid) of invertebrate taxa collected in tundra wetlands near Utqiaġvik, Alaska, summer 2017 and 2018.**

|  |  | **µg C per individual** |
| --- | --- | --- |
| **Taxon** | ***n*** | **(mean ± SE)** |
| Acari | 27 | 24.15 ± 3.08 |
| Crustacea | 74 | 20.57 ± 4.99 |
| Chironomidae | 125 | 80.47 ± 17.54 |
| Plecoptera | 40 | 103.34 ± 19.14 |
| Trichoptera | 65 | 549.08 ± 209.74 |
| Coleoptera | 73 | 261.87 ± 177.02 |
| Tipulidae | 12 | 3196.49 ± 850.28 |
| Oligochaeta | 10 | 1168.27 ± 506.85 |
| Physidae | 86 | 478.39 ± 134.10 |
