# Supplementary material for: Organic matter sources and flows in tundra wetland food webs
Source: PLoS One. 2023 May 26;18(5):e0286368. doi: 10.1371/journal.pone.0286368 (PMC10218757; doi:10.1371/journal.pone.0286368)
Supplement: S4 Table — N/A indicates no data for a given taxon. Numbers of wetlands of each wetland type that were sampled for each organism are in S7 and S8 Tables. (DOCX) [file pone.0286368.s004.docx]

**S4 Table. Mean ± SE of stable isotope values (δ^13^C and δ^15^N) for each invertebrate taxon in different wetland types.** N/A indicates no data for a given taxon. Numbers of wetlands of each wetland type that were sampled for each organism are in Tables S7 and S8.

|  | **Shallow *Arctophila*** | | **Deep *Arctophila*** | | **Shallow *Carex*** | | **Deep *Carex*** | | **Creeks** | | **Deep Open Lakes** | |
| --- | --- | --- | --- | --- | --- | --- | --- | --- | --- | --- | --- | --- |
| Taxon | δ^13^C | δ^15^N | δ^13^C | δ^15^N | δ^13^C | δ^15^N | δ^13^C | δ^15^N | δ^13^C | δ^15^N | δ^13^C | δ^15^N |
| Acari | -34.32 ± 0.20 | 6.73 ± 0.17 | -35.28 ± 0.46 | 6.64 ± 0.25 | -34.45 ± 0.18 | 6.10 ± 0.18 | -32.79 ± 0.19 | 7.45 ± 0.36 | -34.47 | 6.31 | -32.63 | 9.60 |
| Crustacea | -32.95 ± 0.30 | 3.84 ± 0.20 | -33.71 ± 0.12 | 3.77 ± 0.08 | -33.62 ± 0.21 | 3.55 ± 0.19 | -31.26 ± 0.14 | 4.43 ± 0.09 | -33.44 ± 0.56 | 5.59 ± 0.26 | -30.67 ± 0.83 | 10.48 ± 3.07 |
| Chironomidae | -33.36 ± 0.07 | 4.62 ± 0.06 | -33.11 ± 0.08 | 4.02 ± 0.05 | -32.76 ± 0.12 | 4.29 ± 0.08 | -31.64 ± 0.09 | 4.33 ± 0.07 | -32.63 ± 0.25 | 6.67 ± 0.24 | -30.38 ± 0.27 | 8.20 ± 0.09 |
| Plecoptera | -35.33 ± 0.22 | 2.67 ± 0.18 | -34.61 ± 0.27 | 2.64 ± 0.18 | -34.65 ± 0.36 | 2.15 ± 0.15 | -33.64 ± 0.22 | 3.10 ± 0.11 | -35.83 | 1.77 | -31.04 | 6.59 |
| Trichoptera | -33.12 ± 0.15 | 3.21 ± 0.10 | -32.68 ± 0.13 | 3.07 ± 0.09 | -32.59 ± 0.19 | 1.91 ± 0.18 | -31.73 ± 0.08 | 4.27 ± 0.13 | -32.32 ± 0.09 | 3.21 ± 0.75 | -29.88 ± 0.66 | 7.61 ± 0.51 |
| Coleoptera | -33.83 ± 0.10 | 4.00 ± 0.07 | -33.12 ± 0.13 | 4.49 ± 0.08 | -32.95 ± 0.28 | 4.13 ± 0.14 | -31.67 ± 0.13 | 4.42 ± 0.09 | -32.48 ± 0.32 | 4.03 ± 0.20 | -29.89 | 6.27 |
|  |  |  |  |  |  |  |  |  |  |  |  |  |
| Tipulidae | -30.14 ± 0.67 | 3.65 ± 1.14 | -28.84 ± 0.37 | 2.48 ± 0.26 | -29.21 ± 0.18 | 2.65 ± 0.70 | -30.02 ± 0.35 | 1.54 ± 0.04 | N/A | N/A | N/A | N/A |
| Oligochaeta | -27.21 | 2.69 | -30.74 ± 1.07 | 4.41 ± 0.19 | -30.35 ± 1.19 | 3.54 ± 0.47 | -28.15 | 2.41 | -37.51 | 5.88 | -28.33 ± 0.65 | 6.59 ± 0.72 |
| Physidae | -26.11 ± 0.22 | 2.76 ± 0.13 | -27.26 ± 0.17 | 3.25 ± 0.05 | -27.14 ± 0.17 | 3.33 ± 0.08 | -26.32 ± 0.12 | 4.08 ± 0.09 | -27.48 ± 0.41 | 4.03 ± 0.11 | -26.05 | 4.96 |
