# Supplementary material for: Organic matter sources and flows in tundra wetland food webs
Source: PLoS One. 2023 May 26;18(5):e0286368. doi: 10.1371/journal.pone.0286368 (PMC10218757; doi:10.1371/journal.pone.0286368)
Supplement: S5 Table — Gelman-Rubin values substantially greater than 1 indicate lack of model convergence. Gelman-Rubin diagnostics shown are number of parameters created by models, and the number (percentage in parentheses) exceeding thresholds of 1.01, 1.05, and 1.10. Geweke diagnostics are standard z-scores, so 5% of parameters in each chain are expected to exceed ± 1.96, with lower values indicating better model convergence. Geweke values shown in this table are the number of parameters exceeding ± 1.96 (percentage in parentheses). (DOCX) [file pone.0286368.s005.docx]

**S5 Table.** **Diagnostics for MixSIAR Markov-chain Monte Carlo (MCMC) models.** Gelman-Rubin values substantially greater than 1 indicate lack of model convergence. Gelman-Rubin diagnostics shown are number of parameters created by models, and the number (percentage in parentheses) exceeding thresholds of 1.01, 1.05, and 1.10. Geweke diagnostics are standard z-scores, so 5% of parameters in each chain are expected to exceed ± 1.96, with lower values indicating better model convergence. Geweke values shown in this table are the number of parameters exceeding ± 1.96 (percentage in parentheses).

| Model set | | Gelman-Rubin diagnostic | | | Geweke diagnostic | | |
| --- | --- | --- | --- | --- | --- | --- | --- |
| Wetland Type | n parameters | *n* > 1.01 | *n* > 1.05 | *n* > 1.10 | Chain 1 | Chain 2 | Chain 3 |
| Shallow *Arctophila* | 107 | 0 (0) | 0 (0) | 0 (0) | 10 (9.3) | 17 (15.9) | 0 (0) |
| Deep *Arctophila* | 111 | 1 (0.9) | 0 (0) | 0 (0) | 14 (12.6) | 6 (5.4) | 7 (6.3) |
| Shallow *Carex* | 76 | 0 (0) | 0 (0) | 0 (0) | 4 (5.3) | 1 (1.3) | 0 (0) |
| Deep *Carex* | 116 | 0 (0) | 0 (0) | 0 (0) | 22 (19.0) | 0 (0) | 4 (3.4) |
| Stream | 44 | 0 (0) | 0 (0) | 0 (0) | 6 (13.6) | 2 (4.5) | 0 (0) |
| Deep Open Lakes | 42 | 0 (0) | 0 (0) | 0 (0) | 1 (2.3) | 0 (0) | 0 (0) |
