# Supplementary material for: Organic matter sources and flows in tundra wetland food webs
Source: PLoS One. 2023 May 26;18(5):e0286368. doi: 10.1371/journal.pone.0286368 (PMC10218757; doi:10.1371/journal.pone.0286368)
Supplement: S6 Table — (DOCX) [file pone.0286368.s006.docx]

**S6 Table.** **Mean ± SE of mixing model estimates of the percentages of organic matter sources (%A = percentage algae, %PM = percentage peat and macrophytes) for different invertebrate taxa in different wetland types.**

|  | **Shallow *Arctophila*** | | **Deep *Arctophila*** | | **Shallow *Carex*** | | **Deep *Carex*** | | **Streams** | | **Deep Open Lakes** | |
| --- | --- | --- | --- | --- | --- | --- | --- | --- | --- | --- | --- | --- |
| Taxon | %A | %PM | %A | %PM | %A | %PM | %A | %PM | %A | %PM | %A | %PM |
| Acari | 74.7 ± 3.5 | 25.3 ± 3.5 | 81.8 ± 5.7 | 18.2 ± 5.7 | 72.7 ± 4.9 | 27.3 ± 4.9 | 56.9 ± 6.5 | 43.1 ± 6.5 | 70.0 ± 13.3 | 30.0 ± 13.3 | 62.4 ± 20.5 | 37.6 ± 20.5 |
| Crustacea | 63.3 ± 4.1 | 36.7 ± 4.1 | 64.9 ± 2.4 | 35.1 ± 2.4 | 61.4 ± 3.5 | 38.6 ± 3.5 | 38.6 ± 2.0 | 61.4 ± 2.0 | 61.1 ± 6.7 | 38.9 ± 6.7 | 25.8 ± 8.8 | 74.2 ± 8.8 |
| Chironomidae | 58.9 ± 1.7 | 41.1 ± 1.7 | 58.6 ± 1.7 | 41.4 ± 1.7 | 54.5 ± 2.0 | 45.5 ± 2.0 | 40.4 ± 1.4 | 59.6 ± 1.4 | 51.6 ± 3.6 | 48.4 ± 3.6 | 24.5 ± 3.8 | 75.5 ± 3.8 |
| Plecoptera | 76.6 ± 3.8 | 23.4 ± 3.8 | 72.8 ± 4.0 | 27.2 ± 4.0 | 69.3 ± 6.0 | 30.7 ± 6.0 | 63.6 ± 2.8 | 36.4 ± 2.8 | 67.3 ± 19.2 | 32.7 ± 19.2 | 28.1 ± 19.7 | 71.9 ± 19.7 |
| Trichoptera | 59.3 ± 2.6 | 40.7 ± 2.6 | 52.9 ± 2.5 | 47.1 ± 2.5 | 50.0 ± 3.7 | 50.0 ± 3.7 | 38.7 ± 1.9 | 61.3 ± 1.9 | 46.2 ± 9.5 | 53.8 ± 9.5 | 20.3 ± 7.5 | 79.7 ± 7.5 |
| Coleoptera | 62.4 ± 2.3 | 37.6 ± 2.3 | 57.3 ± 2.6 | 42.7 ± 2.6 | 57.9 ± 4.0 | 42.1 ± 4.0 | 39.8 ± 2.0 | 60.2 ± 2.0 | 49.4 ± 4.8 | 50.6 ± 4.8 | 23.3 ± 18.4 | 76.7 ± 18.4 |
