# Supplementary material for: Organic matter sources and flows in tundra wetland food webs
Source: PLoS One. 2023 May 26;18(5):e0286368. doi: 10.1371/journal.pone.0286368 (PMC10218757; doi:10.1371/journal.pone.0286368)
Supplement: S7 Table — Invertebrates were collected near Utqiaġvik, Alaska in summer 2017 and 2018. Means in the same row with the same superscript are not significantly different (PERMANOVA, P > 0.05). N/A indicates no data for a given taxon. (DOCX) [file pone.0286368.s007.docx]

**S7 Table.** **Mean ± SE of biomasses in cores (mg C m^‒2^) of different invertebrate taxa in different tundra wetland types.** Invertebrates were collected near Utqiaġvik, Alaska in summer 2017 and 2018. Means in the same row with the same superscript are not significantly different (PERMANOVA, *P* > 0.05). N/A indicates no data for a given taxon.

_______________________________________________________________________________________________

**mg C / Shallow Deep Shallow Deep Deep Open**

**Taxon indiv *Arctophila Arctophila Carex Carex* Creeks Lakes**

_______________________________________________________________________________________________

*n* of wetlands 14 10 11 7 6 4

Acari 0.03 4.3 ± 1.6^ab^ 1.3 ± 0.7^a^ 6.8 ± 2.4^b^ 0.8 ± 0.5^ab^ 1.2 ± 0.5^ab^ 1.8 ± 1.5^ab^

Crustacea 0.02 14.9 ± 4.3^a^ 39.9 ± 22.6^ab^ 102.1 ± 35.4^b^ 3.3 ± 1.4^a^ 6.2 ± 5.7^a^ 80.2 ± 69.4^ab^

Chironomidae 0.08 167.9 ± 44.6^a^ 141.9 ± 34.5^a^ 261.2 ± 36.2^b^ 162.0 ± 44.6^ac^ 89.0 ± 21.2^c^ 89.0 ± 45.6^c^

Plecoptera 0.12 0.6 ± 0.5^a^ 1.6 ± 1.5^ab^ 12.3 ± 11.7^ab^ 2.2 ± 1.3^ab^ 1.3 ± 1.2^ab^ 5.2 ± 2.6^b^

Trichoptera 0.75 23.6 ± 13.3^a^ N/A 22.6 ± 14.4^a^ 5.9 ± 5.5^a^ N/A 13.8 ± 11.9^a^

Coleoptera 0.28 30.5 ± 15.7^a^ N/A 10.8 ± 6.9^a^ N/A 311.3 ± 284.2^a^ 6.6 ± 5.7^a^

Physidae 0.18 10.3 ± 7.7^a^ 14.4 ± 10.4^a^ 64.4 ± 53.7^a^ N/A 14.0 ± 8.2^a^ N/A

_______________________________________________________________________________________________
