# Supplementary material for: Organic matter sources and flows in tundra wetland food webs
Source: PLoS One. 2023 May 26;18(5):e0286368. doi: 10.1371/journal.pone.0286368 (PMC10218757; doi:10.1371/journal.pone.0286368)
Supplement: S8 Table — Invertebrates were collected near Utqiaġvik, Alaska in summer 2017 and 2018. Means in the same row with the same superscript are not significantly different (PERMANOVA, P > 0.05). N/A indicates no data for a given taxon. (DOCX) [file pone.0286368.s008.docx]

**S8 Table. Mean ± SE of biomasses in net sweeps (mg C m^‒3^) of different invertebrate taxa in different tundra wetland types.** Invertebrates were collected near Utqiaġvik, Alaska in summer 2017 and 2018. Means in the same row with the same superscript are not significantly different (PERMANOVA, *P* > 0.05). N/A indicates no data for a given taxon.

_______________________________________________________________________________________________

**mg C / Shallow Deep Shallow Deep Deep Open**

**Taxon indiv *Arctophila Arctophila Carex Carex* Creeks Lakes**

_______________________________________________________________________________________________

*n* of wetlands 13 10 11 7 6 4

Acari 0.03 8.5 ± 2.83.2^ab^ 2.2 ± 0.6^ab^ 6.1 ± 1.4^b^ 9.1 ± 3.8^b^ 1.6 ± 1.2^a^ 0.9 ± 0.7^a^

Crustacea 0.02 40.2 ± 16.4^ab^ 20.2 ± 6.7^a^ 43.1 ± 21.1^a^ 13.7 ± 4.3^ab^ 5.4 ± 2.8^b^ 31.4 ± 26.2^ab^

Chironomidae 0.08 101.8 ± 16.6^ab^ 93.0 ± 25.7^ab^ 219.1 ± 64.3^a^ 128.8 ± 32.0^ab^ 78.7 ± 21.4^ab^ 46.8 ± 21.5^b^

Plecoptera 0.12 7.5 ± 3.9^a^ 4.7 ± 2.6^a^ 5.7 ± 4.4^a^ 52.3 ± 19.8^b^ 9.3 ± 7.2^a^ 0.5 ± 0.5^a^

Trichoptera 0.75 68.2 ± 25.9^ab^ 18.5 ± 10.9^ab^ 58.6 ± 24.4^ab^ 43.2 ± 11.7^a^ 8.4 ± 3.7^b^ N/A

Coleoptera 0.28 76.2 ± 37.4^a^ 3.7 ± 1.8^b^ 50.3 ± 31.4^a^ 36.2 ± 18.2^a^ 27.6 ± 12.0^a^ 1.3 ± 0.7^c^

Physidae 0.18 20.7 ± 6.9^a^ 14.2 ± 7.4^a^ 72.8 ± 34.3^a^ 12.6 ± 6.5^a^ 35.8 ± 28.3^a^ N/A

_______________________________________________________________________________________________
